# Modulating dream experience: Noninvasive brain stimulation over the sensorimotor cortex reduces dream movement

**DOI:** 10.1038/s41598-020-63479-6

**Published:** 2020-04-21

**Authors:** Valdas Noreika, Jennifer M. Windt, Markus Kern, Katja Valli, Tiina Salonen, Riitta Parkkola, Antti Revonsuo, Ahmed A. Karim, Tonio Ball, Bigna Lenggenhager

**Affiliations:** 1 Department of Psychology, University of Cambridge, CB2 3EB Cambridge, United Kingdom; 2 Department of Psychology and Speech-Language Pathology, University of Turku, 20014 Turku, Finland; 3 Department of Philosophy, Monash University, VIC 3800 Clayton, Australia; 4 Translational Neurotechnology Lab, University of Freiburg, 79106 Freiburg, Germany; 5 Department of Cognitive Neuroscience and Philosophy, University of Skövde, 54128 Skövde, Sweden; 6 Department of Radiology, University and University Hospital of Turku, 20521 Turku, Finland; 7 Department of Psychiatry and Psychotherapy, University of Tübingen, 72076 Tübingen, Germany; 8 Department of Psychology and Neuroscience, Jacobs University, 28759 Bremen, Germany; 9 Department of Health Psychology and Neurorehabilitation, SRH Mobile University, Riedlingen, Germany; 10 Department of Psychology, University of Zurich, 8050 Zurich, Switzerland

**Keywords:** REM sleep, Consciousness

## Abstract

Recently, cortical correlates of specific dream contents have been reported, such as the activation of the sensorimotor cortex during dreamed hand clenching. Yet, despite a close resemblance of such activation patterns to those seen during the corresponding wakeful behaviour, the causal mechanisms underlying specific dream contents remain largely elusive. Here, we aimed to investigate the causal role of the sensorimotor cortex in generating movement and bodily sensations during REM sleep dreaming. Following bihemispheric transcranial direct current stimulation (tDCS) or sham stimulation, guided by functional mapping of the primary motor cortex, naive participants were awakened from REM sleep and responded to a questionnaire on bodily sensations in dreams. Electromyographic (EMG) and electroencephalographic (EEG) recordings were used to quantify physiological changes during the preceding REM period. We found that tDCS, compared to sham stimulation, significantly decreased reports of dream movement, especially of repetitive actions. Other types of bodily experiences, such as tactile or vestibular sensations, were not affected by tDCS, confirming the specificity of stimulation effects to movement sensations. In addition, tDCS reduced EEG interhemispheric coherence in parietal areas and affected the phasic EMG correlation between both arms. These findings show that a complex temporal reorganization of the motor network co-occurred with the reduction of dream movement, revealing a link between central and peripheral motor processes and movement sensations of the dream self. tDCS over the sensorimotor cortex interferes with dream movement during REM sleep, which is consistent with a causal contribution to dream experience and has broader implications for understanding the neural basis of self-experience in dreams.

## Introduction

Dreams are vivid, often emotionally intense and narratively complex experiences occurring in sleep. In our dreams, we feel immersed in alternative worlds and have the experience of interacting with other persons and objects. Often this involves the subjective experience of moving through the dream world, and movement is among the most frequently reported dream experiences, second only to visual imagery^1,2^. Yet these rich subjective experiences stand in stark contrast to the outward unresponsiveness and lack of observable behaviour during sleep. This study aimed to investigate the causal mechanisms underlying dream movement and bodily experience in dreams by applying transcranial direct current stimulation (tDCS) over sensorimotor areas. While most existing studies of the neural underpinnings of bodily experience in dreams and dream movement are correlational, our approach aimed to directly influence dream content and allowed us to speculate on its underlying causes.

Specifically, our goal was to characterize the role of the sensorimotor cortex in the generation of bodily sensations in dreams. We aimed to experimentally modulate motor and other bodily experiences, which are important aspects of self-experience in dreams, through bihemispheric tDCS during REM sleep. After awakening from REM sleep, subjective dream experience was examined through the collection of dream reports and a questionnaire specifically designed to investigate bodily experiences in dreams; neural measures were obtained through electrophysiological sleep data.

This experimental protocol was guided by theoretical and empirical considerations. Our focus on bodily experience was motivated by the centrality of self-experience and subjective presence to dreaming^3–5^. The immersive structure of dreaming is foregrounded in simulation theories^6,7^, in which dreams are described as mental simulations characterized by the experience of a virtual world. Typically, this virtual world is centered on a virtual self and experienced from an internal first-person perspective. The dream self is typically described as actively engaged in dream events, and movement is reported in up to 75% of dreams^5,8^. This immersive *here* and *now* quality, as described in dream reports, is widely regarded as a defining characteristic of dreaming (for discussion and further references, see^7^; for a general defense of the trustworthiness of dream reports, see^9,10^). It is also striking that with few exceptions, both the virtual world and the virtual self in dreams are experienced as real. Simulation views sometimes advocate the idea that “being-in-a-dream” feels the same as “being-in-the-world” during wakefulness^11,12^. Moreover, bodily experience and movement sensations also appear to be central to self-experience and subjective presence during waking^13^, and sensorimotor interaction modulates subjective presence both in real and virtual environments^14^.

Our focus on bodily experience was further guided by findings suggesting high-level activity of the motor cortex during REM sleep^1,15,16^. Generally, REM sleep dreaming has been associated with relative deactivation of executive networks and frontal areas, and with high levels of activity in sensory, motor, and emotional networks as compared to wakefulness^17–19^. Studies focusing on the neural correlates of specific types of bodily dream experiences have shown the sensorimotor cortex to be activated during hand clenching in lucid dreams^20^, and the right superior temporal sulcus, a region involved in the biological motion perception to be activated in dreams with a sense of movement^21^. Furthermore, smooth pursuit eye movements during tracking of a visual target are highly similar during waking perception and lucid REM sleep dreaming^22^. Taken together, these studies suggest a remarkable isomorphism of the neural mechanisms underlying motor control in wakefulness and dreaming. However, the correlative nature of these studies limits their potential to uncover the causal contribution of specific brain regions to dream content.

Older studies attempted to experimentally induce different kinds of dream experience via peripheral and bodily stimulation during sleep. Such causal manipulations that have been shown to have an effect on dream content include vestibular stimulation in rotating chairs^23,24^ or hammocks^25^; light flashes or sprays of water applied to the skin^26^; thermal stimulation^27,28^; tactile stimulation via a blood pressure cuff inflated on the leg^29,30^; and olfactory stimulation^31^. The frequency of stimulus incorporation in dreams is variable and dependent both on the kind of stimulus and the sensory modality. Particularly high incorporation rates were achieved in studies using blood pressure cuff stimulation (40–80%)^29,30^. This method of causally manipulating dream content is promising. However, because the processing of external and peripheral stimuli is attenuated in REM sleep, the precise effect of sensory stimulation on dream content is often nonspecific and unpredictable^32^.

As a more direct method for manipulating dream content that avoids the possibly distorting effect of reduced sensory processing during REM sleep, we previously suggested using tDCS^33,34^. We argued that this method might complement previous attempts to manipulate dream content through sensory and bodily stimulation in sleep (for a clinical and neuroethical discussion of this method, see^34^). Unihemispheric tDCS has been shown to facilitate motor imagery during REM sleep^35^ and to modulate visual imagery during Stage 2 NREM sleep^36^, but not during slow wave sleep^37^ or REM sleep^38^. Furthermore, frontal tDCS increases lucidity in experienced lucid dreamers^39^; and frontal transcranial alternating current stimulation (tACS) increases dissociation, insight and control in novice lucid dreamers^40^. tDCS has also been reported to modulate mind wandering in wakefulness^41^. This is promising, as dreaming has been proposed to be an intensified form of mind wandering, based on phenomenological and neurophysiological similarities^42^.

Here, we applied tDCS over the sensorimotor cortex, aiming to understand its causal role in dream content generation. Since tDCS modulates neural processes associated with motor imagery during wakefulness^43–45^, we expected a similar effect during REM sleep. However, instead of aiming at an overall facilitation of movement sensations in dreams with anodal tDCS^35^, our stimulation protocol was designed to induce selective unilateral changes of motor processing and bodily experience during sleep, which would enable a more focused analysis of the electrophysiological mechanisms underlying dream movement. It is well known that motor effects depend on current direction, with cathodal stimulation being more effective and largely inhibitory and anodal stimulation being less effective and largely facilitatory^46^. We thus adopted a bihemispheric tDCS protocol, expecting to observe an inhibition of bodily experiences on the right side of the body due to cathodal stimulation, and facilitation of bodily experiences on the left side of the body due to anodal stimulation. We hypothesized that if the sensorimotor cortex has a causal role in generating sensorimotor dream content, bihemispheric tDCS over the sensorimotor cortex during REM sleep should modulate movement and other bodily experiences in dreams reported immediately after timed awakenings in the laboratory. To test this hypothesis, we developed a questionnaire focused specifically on bodily sensations in dreams. This questionnaire drew from existing findings on the phenomenology of dreaming^1,2,47^ as well as from findings on bodily experience in altered states and wake-state pathologies^48,49^. It was also inspired by methodological considerations on the comparative usefulness of questionnaires and free dream reports for the study of specific aspects of dream phenomenology, and specifically by several studies on dream emotions^50,51^. These findings guided our focus on certain types of bodily experiences (and in particular movement) as well as our decision to use a questionnaire rather than focus exclusively on the analysis of dream reports (for theoretical discussion of this methodology and further references, see^9,10^). This approach allowed us to probe bodily experiences more systematically than the more common methods of content analysis or quantitative linguistic analysis of dream reports^35^.

Furthermore, we hypothesized that bihemispheric tDCS during REM sleep would interfere with interhemispheric motor networks as well as with spontaneous peripheral muscle activity, which are possible correlates of dream movement. REM sleep is typically characterized by near-complete muscle atonia^52^ and a partial blockade of sensory input^1,53^. At the same time, subtle muscular activity in the form of twitching is frequent in REM sleep and may play a role in the development and maintenance of motor behaviour^54^. A relation to dreaming seems plausible, but remains incompletely understood^55^. To investigate possible effects of bihemispheric tDCS on outward muscular activity, we obtained electromyographic (EMG) measures from both arms.

## Results

### Study outline

**Figure 1:**
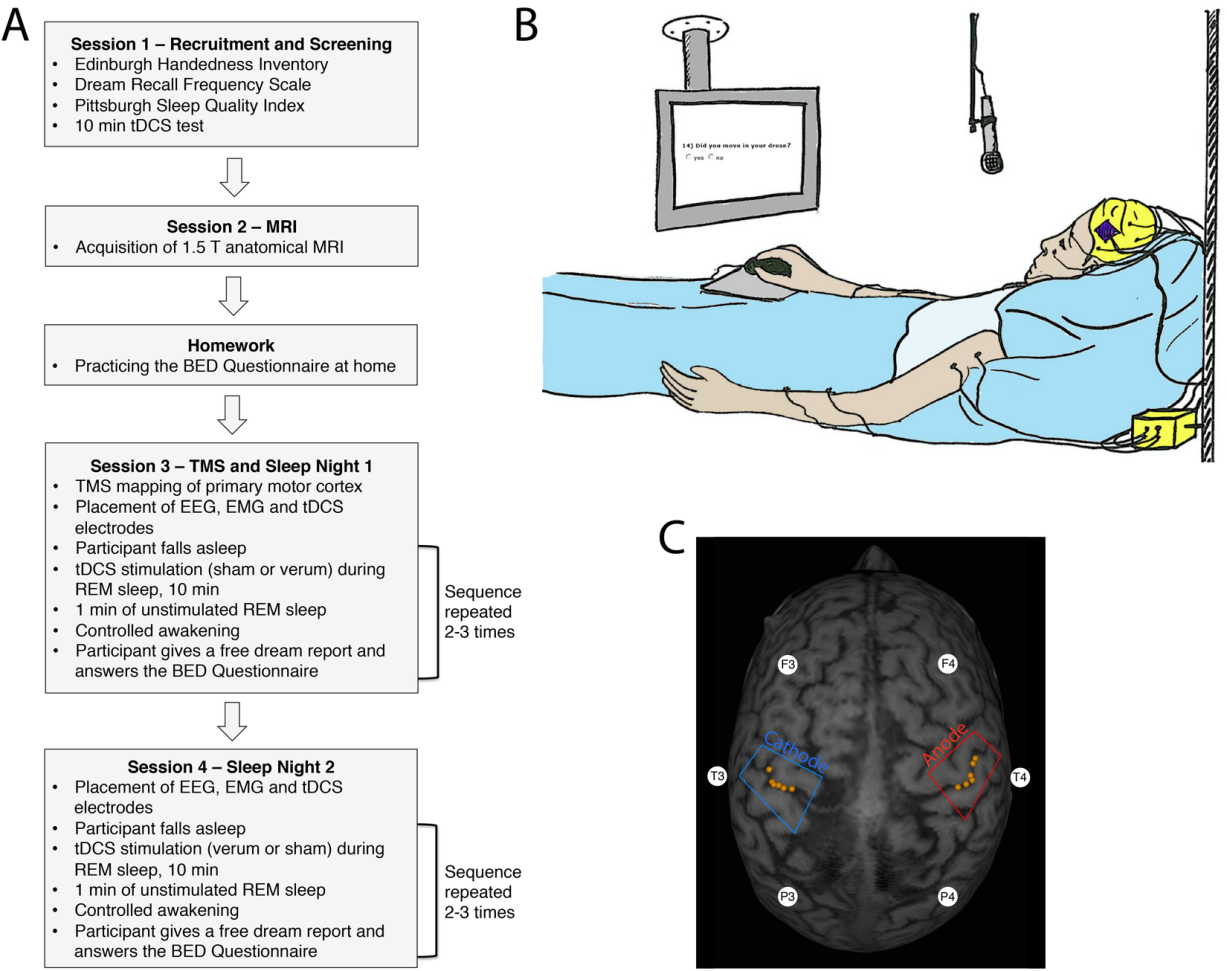
Experimental design. (**A**) Time course of the study. **(B)** Experimental setup during sleep sessions (drawn by Peter Rohner). **(C)** Primary sensorimotor hand areas of a representative participant. Orange dots indicate stimulation sites where TMS pulses in waking participants induced a subjectively experienced hand movement and/or muscle twitch (located approximately at the central sulcus between the somatosensory and somatomotor cortices). The blue box over the left hemisphere represents the cathode tDCS electrode placement site, and the red box over the right hemisphere represents the anode electrode placement site. White circles depict the approximate location of 6 electrodes used for the EEG inter-hemispheric coherence analysis.

The study protocol consisted of a recruitment and screening session, an MRI session, and two sleep sessions on non-consecutive nights (Fig. 1A). In addition, a TMS assessment of motor cortical excitability took place on the evening of the first sleep session. Ten participants were awakened from REM sleep two or three times per night and asked to give free dream reports and answer the Bodily Experiences in Dreams (BED) Questionnaire, which targeted the dream immediately preceding awakening (Fig. 1B). Participants received sham-stimulation during REM sleep on one night and bihemispheric tDCS on the other night. Bihemispheric tDCS montage included a cathode placed over the left sensorimotor cortex and an anode placed over the right sensorimotor cortex (Fig. 1C). In addition to standard polysomnography, central and peripheral electrophysiological data were recorded using 16 electroencephalography (EEG) channels and 4 EMG channels measuring flexor and deltoid muscles in both arms.

### tDCS modulates dream movement

**Figure 2:**
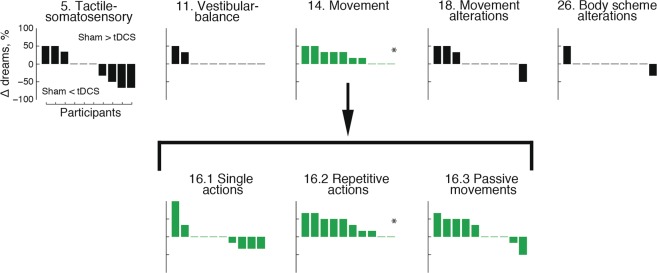
tDCS effects on reported dream experiences. Changes between sham-stimulation and tDCS conditions across the five general categories of dream content (top row) and for particular kinds of movement (bottom row) per participant. Positive and negative values indicate a higher proportion of dreams with a specific experience in the sham-stimulation and tDCS condition, respectively. Individual participants are sorted in descending order beginning with the participant with the highest proportion of dreams with a specific experience in the sham-stimulation condition, compared to the tDCS condition. Participants are sorted separately for each dimension of experience. *p_B_ < 0.05.

The first research question was whether the sensorimotor cortex is involved in the generation of bodily experiences in dreams. To answer this, we compared the percentage of dreams with different types of bodily experiences as reported in the BED Questionnaire between the tDCS and sham stimulation. Among the general dimensions of bodily experience in dreams (tactile/somatosensory, vestibular/balance, movement, movement alterations, body scheme alterations), we found a significant difference only for movement (Fig. 2 and Table 1). Specifically, the proportion of dreams with movement was significantly lower in the tDCS compared to the sham-stimulation condition (paired samples t test: t(9) = 3.77, p_Bx5_ = 0.022, d = 0.85). That is, participants were less likely to answer YES to the question “Did you move in your dream?” when they were awakened 1 min after termination of bihemispheric tDCS. At the individual level, 7 out of 10 participants showed this effect, whereas the remaining 3 participants had equal proportions of dreams with movements between the two conditions (Fig. 2).

**Table 1:** The BED Questionnaire: Percentage of dream reports containing specific bodily experiences following sham-stimulation and tDCS during REM sleep.

Bodily experiences	Sham	tDCS	Statistical test	
	M (SEM)	M (SEM)	t/Z	p_B_
***Five general dimensions***				
5. Tactile-somatosensory	34.9 (12.3)	43.2 (12)	t(9) = 0.59	1
11. Vestibular-balance	8.3 (5.7)	0 (0)	Z = 1.34	0.9
14. Movement	86.6 (7)	63.1 (10.2)	t(9) = 3.77	0.022*
18. Movement alterations	13.3 (6.9)	5 (5)	Z = 0.76	1
26. Body scheme alterations	5 (5)	3.3 (3.3)	Z = 0.45	1
***Movement sub-scales***				
16.1 Single actions	53.3 (13.3)	51.7 (13.3)	Z = 0.22	1
16.2 Repetitive actions	65 (9.8)	30 (8.5)	t(9) = 4.36	0.006*
16.3 Passive movements	30 (8.5)	11.7 (7.9)	t(9) = 1.56	0.45

*Note*. t: paired samples t test; Z: Wilcoxon signed-rank test; *p_B_ < 0.05

To investigate whether specific types of movement were inhibited by tDCS, we compared the proportion of dreams with single actions (i.e. movements that are not repeated immediately after their execution, such as placing a book on the table), repetitive actions (i.e. the same movements repeated several times in a continuous sequence, such as running), and passive movements (i.e. movements determined by external forces, such as traveling by car) between tDCS and sham-stimulation conditions (see Table 2 for examples of movement descriptions in the verbal dream reports). There were significantly less dreams with repetitive actions in the tDCS condition compared to the sham condition (paired samples t test: t(9) = 4.36, p_Bx3_ = 0.006, d = 1.21) (Fig. 2). There were no significant tDCS effects on the frequency of dreams containing single actions or passive movements (see Table 1).

**Table 2:** Examples of different types of movement reported in verbal dream reports.

Movement types	Sham	tDCS
Single actions	“We […] sat down” (P10, N2, A2)	“I was diving” (P1, N1, A1)
	“I hugged her” (P10, N2, A3)	“I was […] to take a pose” (P5, N2, A1)
Repetitive actions	“I remember rubbing quite hard […] my leg” (P1, N2, A1)	“I was swimming in a pool” (P1, N1, A1)
	“I was walking there” (P3, N2, A2)	“I […] was writing something” (P3, N1, A1)
	“we are running away from him” (P4, N2, A1)	“I was cleaning a table” (P5, N2, A2)
	“I had been sleepwalking” (P4, N2, A2)	“I was climbing upstairs” (P7, N2, A2)
	“I was digging the vegetable garden” (P6, N1, A3)	“I was stroking gently [our cat]” (P8, N2, A3)
Passive movements	“we were coming from Lappeenranta with a train” (P4, N2, A1)	“our father was driving me and my brother […] with a car” (P6, N2, A1)
	“they somehow forced [me] to put my hand to fist” (P7, N1, A1)	“he took my hand and pulled me to the middle” (P6, N2, A1)

*Note*. P – participant (1–10), N – night (1–2), A – awakening (1–3).

Interestingly, we found no difference in movement frequency between the stimulation conditions in verbal dream reports that were content analysed by independent judges (see Table S1). This could be due to a considerably smaller proportion of explicitly expressed movements in free reports compared to responses to the BED Questionnaire. Indeed, content analysis indicated a lower proportion of repetitive movements compared to the BED Questionnaire data (Sham stimulation: 38% vs. tDCS: 65%). This is consistent with the possibility that participants tended to omit movements from the spontaneous verbal reports that were given before answering explicit motor questions of the BED Questionnaire (see Supplementary Material), confirming our preference for a questionnaire focused on bodily experiences in this particular study. We also note that we did not instruct participants to focus on bodily experiences in their free dream reports.

According to our questionnaire data, a majority of dream movements involved the whole body (M = 75.5%, SEM = 7.62%) and more rarely the right hand (M = 25%, SEM = 8.23%) or both hands (M = 15.83%, SEM = 7.02%); another unspecified body part was mentioned in only one report. Repetitive actions typically involved the whole body (M = 89.8%, SEM = 6.8%), with only 5.6% of repetitive movements performed by the right hand (Wilcoxon signed-rank test: Z = 2.71, p = 0.007, effect size r = 0.64). Contrary to this, the proportion of single actions was comparable for the whole body (M = 43.8%, SEM = 12.3%) and right hand movements (M = 34.4%, SEM = 11.5%, Wilcoxon signed-rank test: Z = 0.43, p = 0.67, effect size r = 0.11). No systematic body-part or laterality differences were observed between the sham-stimulation and tDCS conditions.

Importantly, the observed reduction of dream movement following tDCS was not related to the overall length of dream reports, which could have been a confounding factor. To test whether the reduction in dream movement was related to shorter dream reports following tDCS, we compared the subjectively estimated duration of dreams during the tDCS and sham-stimulation conditions (BED Questionnaire - Q41: “How long did your dream seem to last, according to subjective experience (in minutes)?”). There was no difference in the subjectively reported duration of dream reports between tDCS (Median = 9.17 min, range from 1.5 min to 97.5 min) and sham-stimulation (Median = 9.67 min, range from 0.83 min to 40 min) conditions (Wilcoxon signed-rank test: Z = 0.36, p = 0.72, r = 0.11). Furthermore, we compared the word count of dream reports. Once again, there was no significant difference between tDCS (M = 76.1, SEM = 16.31) and sham-stimulation (M = 124.2, SEM = 34.68) conditions (paired samples t test: t(9) = 1.69, p = 0.124, d = 0.56, Bf in favor of the null = 1.11). On four occasions, participants remembered and reported additional details of a dream after completing the original dream report and questionnaire, while they were trying to fall asleep again. When these secondary reports were included in the word count analysis, there was still no significant difference in word count between tDCS (M = 89, SEM = 19.83) and sham-stimulation (M = 124.98, SEM = 34.55) conditions (paired samples t test: t(9) = 1.15, p = 0.281, d = 0.4, Bf in favor of the null = 1.91). We thus conclude that differences in the length of dream reports (and in the subjectively estimated duration of dreams) were not related to the observed reduction of dream movement following tDCS.

To assess the possible effect of the time of night, we compared the answers to the five main questions (tactile-somatosensory, vestibular-balance, movement, movement alterations, body scheme alterations) between the first and the last reports of each night, separately for the sham-stimulation and tDCS conditions (see Supplementary Material and Table S2). No systematic differences were observed, excluding cumulative effects over night.

### tDCS modulation of EEG activity

Given the opposing directions of bihemispheric tDCS in the current study, i.e. an expected cathodal inhibitory effect over the left motor cortex and an anodal excitatory effect over the right motor cortex, we hypothesized that a reduction of repetitive whole-body actions in response to tDCS was due to a decreased inter-hemispheric coordination of motor processing. To investigate this hypothesis, we restricted EEG analysis to the beta frequency band, because (1) transient and tonic changes in EEG beta oscillatory activity underlie cortical processing of both real^56–58^ and imagined^59,60^ movements; (2) preparation and execution of movement involves inter-hemispheric functional coupling in the beta frequency range^61,62^; and (3) motor impairment and successful rehabilitation involve changes in the inter-hemispheric interaction in the beta frequency range^63,64^. We thus expected bihemispheric tDCS to destabilize motor processing by reducing inter-hemispheric coherence in the beta frequency range.

**Figure 3:**
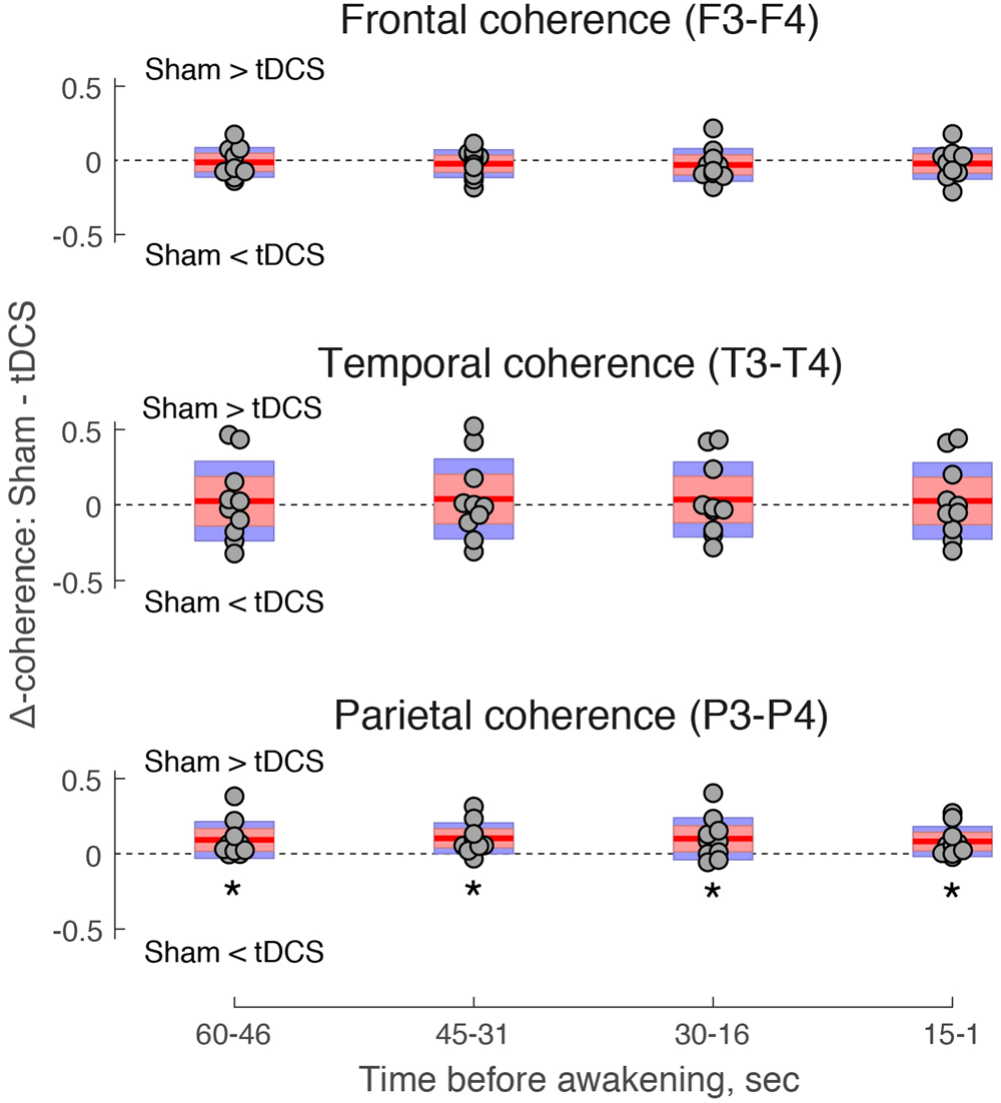
EEG coherence following tDCS during REM sleep. Inter-hemispheric EEG coherence between frontal (top), temporal (middle), and parietal (bottom) electrodes surrounding the tDCS site, expressed as a difference between sham-stimulation and tDCS conditions (Δ-coherence). Jittered circles represent individual participants. Red lines depict the mean of Δ-coherence, pink bars represent 1 standard deviation (SD), and blue bars represent 95% confidence intervals for the mean. Positive values indicate higher coherence in the sham-stimulation condition, whereas negative values indicate higher coherence in the tDCS condition. Δ-coherence is plotted separately in four stimulation-free time intervals preceding controlled awakenings from REM sleep. In the parietal region, coherence was reduced by tDCS compared to sham stimulation in −60- to 46 sec (Z = 2.5, p = 0.013, r = 0.79), −45 to −31 sec (t(9) = 3.17, p = 0.011, d = 0.97), −30 to −16 sec (t(9) = 2.27, p = 0.05, d = 0.88) and −15 to −1 sec (t(9) = 2.57, p = 0.03, d = 0.74) time intervals. * p < 0.05.

As predicted, we observed a significant decrease in coherence between parietal electrodes P3-P4 following tDCS compared to sham-stimulation during a 1-minute stimulation-free period before awakening (Wilcoxon signed-rank test: Z = 2.5, p_Bx3_ = 0.039, effect size r = 0.79). No inter-hemispheric tDCS effects were observed between frontal (paired samples t test: t(9) = 0.72, p_Bx3_ = 1, d = 0.244) or temporal electrodes (t(9) = 0.38, p_Bx3_ = 1, d = 0.114). To control for the temporal specificity of the decrease of parietal coherence, we repeated the same analysis in four separate time intervals following the termination of stimulation: −60 to −46 sec, −45 to −31 sec, −30 to −16 sec, and −15 sec to −1 sec prior to awakening. A significant effect observed only in the time window before awakening (i.e. −30 to −16 sec, and/or −15 sec to −1 sec) would indicate a non-specific effect of experimental stimulation. Compared to sham-stimulation, a significant decrease of parietal coherence took place in the tDCS condition throughout all four sub-intervals between the offset of stimulation and the onset of awakening, confirming a direct and relatively long-lasting tDCS effect on parietal coherence in the beta-frequency range (Fig. 3).

Given that EEG coherence can be affected by spectral power differences between conditions^65^, we carried out a control analysis to compare beta power in the electrodes adjacent to the stimulation site across a 1 min stimulation-free pre-awakening period. There was a significant decrease of beta power at the left parietal site (P3) in the tDCS compared to the sham-stimulation condition (paired samples t test: t(9) = 2.29, p = 0.048, d = 0.37, Bf in favor of the null = 0.64), whereas tDCS did not modulate beta power in the right parietal site (P4) (t(9) = 0.73, p = 0.48, d = 0.088, Bf in favor of the null = 2.93). The observed trend was investigated further across four 15 sec sub-intervals. No tDCS effects were observed regarding beta power in P3 electrode during time intervals immediately following motor cortex stimulation, i.e. −60 to −46 sec (paired samples t test: t(9) = 1.159, p = 0.276, d = 0.25, Bf in favor of the null = 1.89) and −45 to −31 sec (t(9) = 1.172, p  =  0.271, d = 0.3, Bf in favor of the null=1.87). Contrary to this, beta power decreased during time intervals preceding awakenings: −30 to −16 sec (paired samples t test: t(9) = 2.433, p  =  0.038, d = 0.433, Bf in favor of the null = 0.46) and −15 to −1 sec (t(9) = 2.829, p  =  0.02, d  = 0.379, Bf in favor of the null = 0.28). Given that EEG beta coherence was modulated by tDCS across all four time intervals, we conclude that its decrease was not due to the temporally constricted changes in beta spectral power.

Exploratory analysis of inter-hemispheric EEG coherence across a wider range of frequencies (2–45 Hz) indicated that the tDCS-driven decrease of coherence was maximal in the high beta frequency range (21–31 Hz) (see Supplementary Material and Fig. S1). In addition, a small effect of decreasing coherence was observed in the low gamma frequency range (37–43 Hz) immediately following tDCS, i.e. −60 to −31 sec before awakenings (see Supplementary Material and Fig. S2). There was no significant tDCS effect in the delta, theta, alpha and low beta frequency ranges (2–18 Hz).

### tDCS modulation of EMG activity

**Figure 4:**
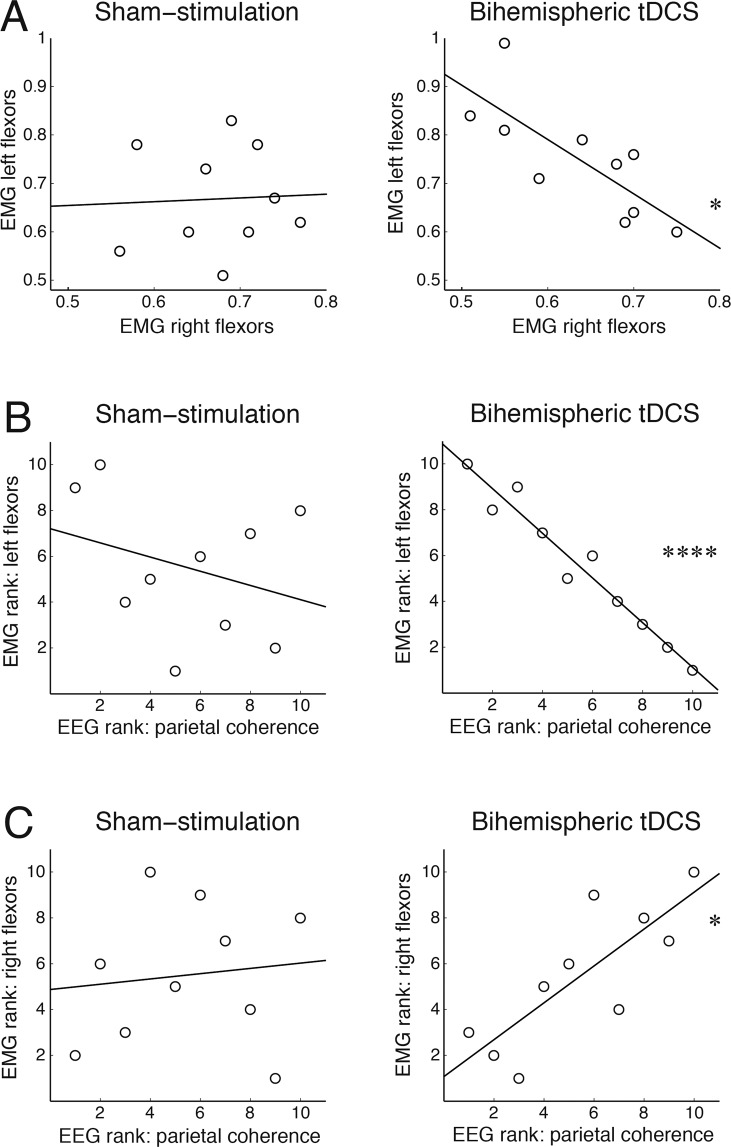
Bihemispheric tDCS during REM sleep modulates phasic activity of the forearm muscles. (**A**) Correlation of EMG shift towards phasic activity between the left and right forearm flexor muscles in the sham-stimulation and tDCS conditions. **(B,C)** Correlation between EMG shift towards phasic activity and EEG parietal coherence in the beta frequency band, plotted separately for the left and right forearm recordings, in the sham-stimulation and tDCS conditions. Ranked data are plotted in **(B,C)** as Spearman’s rank order correlations were carried between EMG and EEG measures. In all plots, the least-squares lines are plotted to visualize associations between variables. *p_B_ < 0.05, ****p_B_ < 0.00005.

We observed a significant association in the proportion of phasic EMG activity in the flexors between the left and right arms during the 1-min period of REM sleep from the offset of tDCS to the controlled awakening (Pearson correlation: forearm flexors: r = −0.769, p_Bx4_ = 0.037; deltoids: r = −738, p_Bx4_ = 0.06). The negative correlation between the arms likely reflects the asymmetrical modality of stimulation with the cathode placed over the right sensorimotor cortex and the anode over the left sensorimotor cortex. Contrary to this, there was no association in the proportion of phasic EMG between forearms following sham stimulation (Pearson correlation: forearm flexors: r = 0.095, p_Bx4_ = 1; deltoids: r = 0.308, p_Bx4_ = 1), indicating that muscle activity varied independently (Fig. 4A). Regarding absolute EMG values, there was no difference between phasic activity in the left as compared to the right arm in either the sham-stimulation condition (paired samples t test: forearm flexors: t(9) = 0.12, p_Bx4_ = 1, d = 0.08; deltoids: t(9) = 1.52, p_Bx4_ = 0.66, d = 0.57) of following tDCS (forearm flexors: t(9) = 1.88, p_Bx4_ = 0.37, d = 1.13; deltoids: t(9) = 1.96, p_Bx4_ = 0.33, d = 1.08).

Next, we investigated whether peripheral EMG activity is associated with EEG parietal coherence in the beta frequency band, which decreased in response to tDCS during REM sleep (Fig. 4B,C). In the tDCS condition, EEG coherence was significantly associated with the proportion of phasic activity in the left flexor muscles (Spearman rank order correlation: rho = −0.976, p_Bx8_ = 0.00001) and the right flexor muscles (rho=0.806, p_Bx8_ = 0.039). Interestingly, higher parietal coherence was associated with a larger proportion of phasic activity in the right forearm muscles and a lower proportion of phasic activity in the left forearm muscles, once again likely reflecting the differential effects of anodal vs. cathodal stimulation. No association was observed between parietal EEG coherence and the proportion of phasic activity in flexor muscles in the sham stimulation condition (lowest p_Bx8_ = 1). Likewise, there was no association between parietal EEG coherence and deltoid EMG during either sham-stimulation (lowest p_Bx8_ = 1) or tDCS conditions (lowest p_Bx8_ = 0.72), indicating a site-specific interaction between EEG and EMG measures.

## Discussion

The foremost aim of our study was to investigate the role of the sensorimotor cortex in generating bodily sensations in REM sleep dreams by modulating the excitability of the sensorimotor cortex with tDCS. We found that compared to sham stimulation, bihemispheric tDCS over the sensorimotor cortex reduced specifically the frequency of repetitive actions of the dream self in preceding REM sleep dreams, as measured through responses to the BED Questionnaire. This finding supports the claim that the sensorimotor cortex is causally involved in the generation of dream movement. Even though we did not observe hypothesized laterality differences in dream movement, i.e. inhibition on the right side of body and facilitation on the left side of body, tDCS interfered with inter-hemispheric EEG coherence and peripheral EMG activity, pointing to a change in both the central and peripheral motor systems in response to bihemispheric tDCS during REM sleep.

### Frequency of bodily sensations and movement in dreams

To systematically assess bodily sensations in dreams, we developed a questionnaire designed to capture various dimensions of bodily experiences in dreams (see Table 2 for the exemplary questions). Interestingly, independently of tDCS, our data suggest that while dream movements were very common, other bodily sensations such as somatosensory sensations, vestibular sensations or body scheme alterations were rather rare. This overall pattern of frequent dream movement coupled with rare reports of other bodily sensations has been found in previous studies^1,47,55^. Our study extends the previous work largely based on free dream reports by showing that when different types of bodily experiences are specifically investigated through the use of a questionnaire, movements and tactile sensations remain the predominant dimensions of bodily experience in dreams. Thus, content analysis- and questionnaire-based studies generally provide converging evidence for the important role of sensorimotor phenomena in dreams.

However, our data also suggest that where more specific questions about dream phenomenology are concerned, results from content analysis of free dream reports and questionnaire data may diverge. In our study, the results from content analysis of movements in free dream reports were broadly consistent with those from the questionnaire. However, the modulatory effect of tDCS on dream content involving a reduction of repetitive movements was only found in the questionnaire data but not in the dream reports. Speculating that specific aspects of dream phenomenology, including specific bodily experiences, may remain unreported unless explicitly probed, we expected that responses to the questionnaire would provide a more accurate representation of bodily experience in dreams than the one we could infer from dream content analysis.

Indeed, similar differences between questionnaire results and dream report analyses have also been found for emotions. The frequency of emotions increases 10-fold if participants are asked to report emotions on a line-by-line basis, as compared to free dream reports^50^. When participants are asked to rate the kinds of emotions experienced in their dreams, they specifically report more positive emotions than are found when their dream reports are analyzed by independent judges^51,66^. This discrepancy raises important methodological issues that to date have not been fully resolved, and both methods likely have weaknesses and suffer from different kinds of bias^51^. One reason for the discrepancy, however, could be that free dream reports lack the focus to allow independent judges to pick up on specific aspects of dream phenomenology, such as emotions or movements. By contrast, when participants’ focus is directed to these aspects, such as through the use of questionnaires, this leads to more precise reporting. In our data, similar proportions of different types of movements between external ratings and questionnaire responses, together with the fact that movements were reported more frequently in the questionnaire data, make us lean towards this interpretation. There are also likely differences in what is reported: in free dream reports, individual movements need to be described in some detail for them to be rated by external judges. By contrast, in the questionnaire, participants rate the occurrence and frequency of specific movement types over the entire dream. Again, this may lead to a more comprehensive picture, but also bears the danger of overgeneralizing.

In sum, while claims about the accuracy of subjective dream data, including questionnaires, are notoriously hard to assess, the assumption that detailed questionnaires are a more sensitive measure of specific aspects of dream phenomenology than content analysis of free reports is consistent with our findings. Specifically, while the overall pattern of bodily experience seemed similar in free reports and in the questionnaire responses, the latter indicated more frequent bodily experiences, allowing for a more detailed analysis. The predominance of dream movement in our data also seems to be in line with a recent suggestion that kinesthesia is central to the generation of dream experience, at least during sleep onset^67^. At the same time, in our study, 36.9% of dream reports following tDCS contained no movements. It therefore seems that self-movements are not strictly necessary to sustain REM sleep dreaming. Moreover, the decrease of dream movement did not reduce the length of dream reports in our sample. Whether these dreams still involved e.g. observed movement is an open question.

### Electrophysiological effects of bihemispheric tDCS

Bihemispheric tDCS over the sensorimotor cortex, as compared to sham stimulation, specifically altered repetitive actions in dreams. Repetitive actions are typically dependent on implicit memory of learnt motor sequences (e.g., walking), the automatic processing of which does not require explicit awareness and monitoring of movements. Such learnt, automatic movements, as compared to more controlled and deliberate movements, are also associated with a smaller increase of activity in brain areas related to motor processing^68^. Thus, a relatively modest tDCS interference with cortical processing arguably might have down-regulated motor cortex activity involved in the processing of automatic movements, reducing it to the baseline resting level and simultaneously inhibiting the occurrence of repetitive actions in dreams. Contrary to this, the relatively stronger cortical activation underlying single controlled actions might not have been reduced sufficiently by tDCS interference to significantly alter dream content. This would explain why our results showed a specific decrease in repetitive actions, while the frequency of single actions in dreams remained relatively high during tDCS and did not significantly differ from sham stimulation. Alternatively, bihemispheric stimulation might have interfered with the temporal coordination of dream movement, prohibiting long sequences of repetitive actions, but sparing temporally restricted single actions. Indeed, dream imagery is notoriously unstable and prone to change in discontinuous jumps^69^. Such possibilities should be more directly assessed in future studies, e.g. using motor imagery tasks during wakefulness that would allow for a more stringent control of movement complexity.

We found that bihemispheric tDCS interfered with neural processing in the beta frequency band, classically linked to motor processing^56–64,70^. In our setup, bihemispheric tDCS reduced inter-hemispheric coherence of parietal beta oscillations. Arguably, the differential montage of tDCS electrodes, i.e. the excitatory anode over the right sensorimotor cortex and the inhibitory cathode over the left sensorimotor cortex, disrupted inter-hemispheric coordination of motor commands, reducing the rate of repetitive actions associated with whole body movements in dreams. A differential effect of bihemispheric tDCS was also observed in the phasic EMG activity of the arm muscles. While phasic EMG varied independently between the arms during sham stimulation, a strong negative correlation was observed following tDCS, i.e. it suppressed phasic muscle activity in one arm while increasing it in the other arm.

We expected that such destabilizing and hemisphere-specific effects of tDCS would also cause unilateral distortions, i.e. inhibition vs facilitation, of bodily sensations in dreams. However, the observed reduction of dream movement was independent of the laterality of stimulation. That is, the decrease of inter-hemispheric EEG coherence and the emergence of phasic EMG anticorrelation between arms did not translate into unilateral effects on the dream body. We can only speculate on the lack of laterality effects, and further studies will be important to understand the underlying mechanisms. First, it is possible that the bihemispheric tDCS protocol we adopted inhibited cortical and cerebellar motor networks bilaterally, an effect previously reported in the resting state neuroimaging studies^71,72^. Second, it is conceivable that bihemispheric tDCS produced hemisphere-specific facilitation vs. inhibition of motor processing, and such asymmetry resulted in the reduction of repetitive dream movements that depend on the coordinated whole-body performance. Our EEG and EMG findings, i.e. reduction of EEG inter-hemispheric coherence and negative correlation of phasic EMG between arms, support the latter interpretation.

To detect effects on other modalities (e.g. body image distortion, vestibular sensations), a larger group of participants might be necessary. Moreover, the absence of modulatory effects of tDCS on somatosensory experiences, which were reported quite frequently by our participants, could be related to the placement of the tDCS electrodes that was specifically determined by the location of the hand area in the primary motor cortex.

### Implications for consciousness studies

Our study suggests a methodology for identifying, via causal manipulation, the neural correlates of specific types of dream experience. Thus, beyond dream and sleep research, our findings also have more general implications for consciousness research. First, they add another piece of evidence that the neural correlates of specific dream content match the neural correlates of corresponding cognitive and behavioural functions during wakefulness^21^. Going beyond mere correlation, our results allow us to speculate that the motor cortex might indeed be involved in the generation of movement sensations in dreams.

Our results also shed light on the phenomenological profile of self-representation in dreams. In simulation theories, the subjective sense of presence, or the experience of a self in a world, is central to dreaming. While this highlights the importance of self-simulation, the precise pattern of self-experience in dreams, as compared to wakefulness, raises questions^10^. One possibility is that bodily experience in dreams replicates waking experience; another is that dreams are characterized by a comparative overrepresentation of movement and an underrepresentation of other types of bodily experience (e.g. tactile, thermal, or pain sensations). Our finding that tDCS selectively altered dream movement, taken together with the comparatively low frequency of other types of bodily experience in dreams, is consistent with the second possibility. Future studies could aim to further investigate this question by systematically comparing questionnaire data and reports of bodily experience in both dreams and wakefulness.

A related question concerns the relation between bodily experiences in dreams and the sleeping physical body. It is commonly thought that dream experience, including bodily experience, is completely independent of outward muscular activity and stimulation of the physical body; this is reflected in the idea that dreaming is isolated from external and bodily stimuli^12^ and that REM sleep is characterized by the sensory input/motor output blockade^2^. However, there are empirical and theoretical reasons for thinking that varying degrees of concordance between dream experience and the physical body exist, on both the levels of sensory input and motor output^55,73^. Lesion studies in cats have shown that pontine lesions, which eliminate REM-sleep related muscular atonia, induce organized motor behavior, such as searching and attacking, during REM sleep^74,75^, possibly indicating dream behaviours. Further examples include (illusory) own-body perception, such as when stimulation to the sleeping body is incorporated in dreams^29,30^, and dream enactment behaviors in humans, in which outward muscular activity corresponds to movement sensations in dreams. REM sleep behavior disorder, in which seemingly goal-directed behaviors during REM sleep (such as attacking one’s sleeping partner, attempting to run, etc.) match subjective dream reports, is an extreme example^76–78^. But REM sleep is also accompanied by subtler muscular activity in the form of twitching^79^. Its concordance with dream experience seems plausible but has not been systematically investigated.

In our study, bihemispheric tDCS during REM sleep modulated not only dream movement but also outward muscular activity in the arms. Due to the absence of movement reports in several participants, we could not reliably relate individual variance in subjective movement reports to electrophysiological measures. However, our findings are consistent with the possibility that changes in dream movement are related to changes in outward muscular activity during REM sleep. A promising avenue for future research could be to investigate the relevance of bihemispheric tDCS for several movement-related sleep disorders. REM sleep behaviour disorder would be a good place to start because of the match between dream movements and outward physical activity. Other disorders that could potentially benefit from the inhibition of motor activity include sleepwalking and periodic limb movement disorder. Here, however, the association with dream experience is less clear and should be investigated more directly. Furthermore, as these are typically NREM sleep disorders, it is not certain whether neurophysiological effects of tDCS would be comparable to those observed in the present study of REM sleep.

### Limitations and outlook

Despite these promising results, the current study has several limitations. First, the effects of tDCS on mental states have been repetitively challenged by replicability difficulties^80–82^ and should thus be treated with caution. Nevertheless, given that motor cortex tDCS during wakefulness provides the most reliable effects^81,83^, we expect the same to hold during REM sleep. Second, due to the very complicated and time-intensive protocol of the study, we could only recruit a rather small number of participants. Thus, larger samples and replication studies will be needed in future^84^. Furthermore, and again due to the complexity of the setup, we did not include a control stimulation site nor did we switch the side of the bihemispheric stimulation (to left anodal, right cathodal stimulation), which would be especially interesting to disentangle hemisphere-specific effects. Future studies with a larger sample of participants should also explore whether bihemispheric tDCS during REM sleep interferes with a wider range of EEG frequencies involved in motor processing, including alpha and gamma bands as well as broadband responses^85,86^. Last but not least, our data suggest that the questionnaire that we developed is more sensitive to the tDCS induced changes than free dream reports; this is in line with findings that questionnaires are generally more sensitive to details of dream phenomenology (such as emotions) than free reports^50,51,66,87^. While the questionnaire was developed by the authors and has not yet been validated, it is open to future refinement and this would be a valuable next step.

## Conclusions

To conclude, this study provided, in a controlled setup, evidence that stimulation over the sensorimotor cortex modulates dream content in healthy participants during REM sleep. This has important implications for various research fields, including consciousness research and sleep and dream research. Future studies will have to pinpoint more specifically which neural mechanisms underlie the inhibition of repetitive movements of the dream self and whether the observed subjective and neurophysiological effects are sufficiently long-lasting to warrant clinical studies in, for example, parasomnia patients.

## Methods

### Participants

Aiming to recruit 10 right-handed individuals with high dream recall frequency and good sleep quality, potential participants were screened with the Edinburgh Handedness Inventory^88^ and the Dream Recall Frequency (DRF) scale^89^, which assesses the frequency with which people are able to remember dreams at home, and the Pittsburgh Sleep Quality Index (PSQI)^90^. The DRF scale consists of a single question “How often do you remember your dreams?” and 7 possible answers: 0 = never, 1 = less than once a month, 2 = about once a month, 3 = twice or three times a month, 4 = about once a week, 5 = several times a week, and 6 = almost every morning. Poor dream recallers were excluded from the study. This focus on high recallers (defined in our study as DRF responses 4–6) is common in laboratory studies of dreaming and is important to ensure feasibility, as it maximizes the number of reports. In addition, aiming to assess personal interest and motivation, we asked potential participants “Are you generally interested in dreams?” with 5 possible answers: 1 = not at all, 2 = mainly uninterested, 3 = in-between, 4 = mainly interested, and 5 = very interested.

Furthermore, we aimed to recruit individuals whose global PSQI score did not exceed 4 (with 0 indicating no sleep difficulty and 21 indicating severe difficulties in sleep) and whose sleep latency score indicated they typically needed less than 30 minutes to fall asleep. Again, this was important to ensure feasibility and to maximize awakenings with dream recall.

Given that the application of tDCS may occasionally induce itching, tickling, heat sensations under the electrodes, or even a temporary headache^91^, we introduced potential participants to the tDCS technique before they made their final commitment to take part in the study. After screening for MRI and tDCS contraindications, they were given the opportunity to familiarize themselves with the tDCS procedure before spending their first night in the laboratory. Participants were stimulated for 10 min with tDCS of 1 mA current over the C3 and C4 electrode sites according to the 10–20 EEG system (approximately over the sensorimotor cortex), which helped them decide whether they wanted to participate in the actual experiment. This also helped minimize the risk that tDCS during REM sleep would lead to awakening.

After screening 16 potential participants, we were able to recruit 10 healthy right-handed university students (4 men and 6 women, mean age 26.8, range 4.4 years). The mean handedness index was 0.9 (SD = 0.11; range 0.73 to 1). The mean DRF score was 5.4 (SD  =  0.79, Min = 4, Max = 6), indicating high spontaneous dream recall. While this might introduce bias towards high recallers’ dreams, it is arguably the most feasible recruitment strategy for a costly and time-consuming sleep laboratory study. Regarding personal interest in dreams, two participants responded “in-between”, seven participants were “mainly interested”, and one participant was “very interested”, indicating high motivation to take part in the study. All participants gave their written informed consent according to the Declaration of Helsinki. All experimental protocols of the study were approved by the Ethics Committee of the Hospital District of Southwest Finland, and the study was carried out in accordance with the relevant guidelines and regulations. Participants were financially compensated with 40 euros per night and 10 euros per hour for daytime testing.

### MRI-TMS mapping of the primary sensorimotor hand area

ECoG measurement of the electric field induced by tDCS in human patients with refractory epilepsy as well as computational modelling of tDCS effects in healthy participants suggest that the spatial focality of tDCS decreases if stimulation electrodes are misplaced by >1 cm^92^. Thus, aiming to constrain between-participant variance of the stimulation focus below 1 cm, the location of the hand area in the primary sensorimotor cortex in both hemispheres was determined individually for each participant with the help of magnetic resonance imaging (MRI) and transcranial magnetic stimulation (TMS). Anatomical brain images were acquired with a 1.5 T MRI scanner Philips Intera at the Turku PET Centre. 3D models of the brain were created using 3D T1-weighted MR sequence. A hospital radiologist confirmed that the brain MRI was normal in all cases. Afterwards, the approximate location of primary sensorimotor hand representations was visually determined from anatomical brain images based on macro-anatomical landmarks^93^.

Based on this analysis, the location of the primary sensorimotor hand area was determined for each participant in a separate TMS session, which was carried out on the evening of the first experimental night at the Department of Psychology at the University of Turku. TMS pulses were delivered using eXimia™ TMS stimulator with NBS navigation system (Nexstim Ltd., Helsinki, Finland), which allowed us to navigate within individual anatomical MRI with an approximately 6-mm spatial resolution containing all sources of errors^94^. Participants sat on a reclining chair with their eyes closed and both arms supported by a pillow to ensure that their arm muscles were relaxed. TMS was carried out in a single pulse mode using a figure-of-eight-shaped coil that was held tangentially against the participant’s head. The current direction of the second phase of the biphasic pulse was oriented perpendicularly to the post-central gyrus in the posterior to the anterior direction at the bank between pre-central and post-central sulci^95^ (Fig. 1C).

First, a rough location of the hand area was estimated by asking participants to report whether they experienced any hand movement following a TMS pulse over the motor cortex in the contralateral hemisphere. Once a reliable hotspot was found, an individual motor threshold, i.e. the minimum TMS intensity required to induce the subjective experience of a hand movement, was determined with the maximum likelihood threshold hunting (MLTH) procedure^96^. In this process, 20 pulses were delivered to the hand area with different stimulus intensities, starting at 60% of maximal TMS intensity. The mean motor threshold was 56.1% (SD = 12.4, Min = 28, Max = 76.7) of maximal TMS intensity for the left hemisphere, and 59.2% (SD = 16.5, Min = 24.8, Max = 77.12) for the right hemisphere. While motor thresholds did not differ systematically between the hemispheres (paired samples t test: t(9) = 1.02, p = 0.34, Bf in favor of the null = 2.2), there was a strong inter-hemispheric correlation of motor thresholds (Pearson correlation: r = 0.82, p = 0.004).

Following estimation of individual motor thresholds, the most ventral and caudal points of the hand representation in the primary motor cortex were estimated by delivering TMS pulses with the intensity of 10% above the level of the individual motor threshold. This procedure was consecutively performed for both hemispheres, yielding bilateral hand representation maps that were later used to place tDCS electrodes (Fig. 1C).

### tDCS over the primary sensorimotor cortex during REM sleep

tDCS and sham-stimulation sessions were conducted in the Sleep Laboratory of the Centre for Cognitive Neuroscience at the University of Turku over two non-consecutive nights with each participant. Microprocessor-controlled programmable 1-channel Eldith DC-Stimulator PLUS (Electro-Diagnostic & Therapeutic Systems GmbH, Ilmenau, Germany) was used as a stimulation device.

tDCS was applied bilaterally to hand areas in order to modulate the excitability level of the primary sensorimotor cortex during REM sleep. Participants were asked to avoid caffeine for 6 hours and alcohol and other CNS-affecting drugs for 24 hours prior to the experiment. To ensure these requirements were met, participants filled out the custom-made Pre-Sleep Questionnaire before each session. For each participant, the two stimulation sessions were separated by at least one week in order to avoid interference effects.

Two 35 cm^2^ sized sponge-covered rubber electrodes were soaked with water, and Ten20 electrode paste (Weaver and Company) was applied on both sides of the sponge. The electrodes were placed bilaterally along the central sulcus posterior to the primary motor hand areas, which were determined with the help of MRI-guided TMS (Fig. 1C). They were supported with a comfortable bandage throughout the night. The impedance of tDCS electrodes was kept under 5 kΩ. tDCS was carried out on one experimental night and sham-stimulation took place on another night. Participants were blind to the experimental conditions, i.e. whether the tDCS session was followed by the sham session (N = 5) or vice versa. An equal number of participants was assigned randomly to each condition.

We also considered a double-blind design when planning the study. However, this was not practicable, as experimenters had to monitor EEG throughout stimulation in order to be certain whether REM sleep was continuous until the point of awakening. Given that tDCS induces distinctive EEG artefacts during current ramp up and ramp down periods, the experimental condition would always be revealed to experimenters.

During the tDCS night, 1 mA electric current was delivered to participants‘ scalp two or three times per night for 10 min during REM sleep, starting with the second sleep cycle. To minimize the possibility of subjective sensations of stimulation, such as tingling and itching, the current was ramped up and ramped down over 8 sec periods. It has been reported that tDCS-induced neuroplasticity may accumulate over time^97^, and it is conceivable that stimulation in one REM sleep cycle may have an effect on subsequent REM sleep cycles. In order to keep the stimulation effects consistent throughout the night, we did not mix tDCS and sham-stimulation conditions within a single night. In addition, the electrode over the right sensorimotor area was always the anode, and the electrode over the left sensorimotor area was always the cathode. This procedure ensured that the asymmetric stimulation during one sleep cycle would not interfere with or cancel stimulation effects during another cycle. We chose to place the cathode over the dominant left hemisphere with the aim to disrupt dream movements on the dominant right side of body.

During the sham-stimulation night, stimulation was conducted by switching on the DC device, ramping the current up to 1 mA over an 8 sec period, then stimulating only for a few seconds (M = 5.8 sec, SEM = 0.48), and finally ramping the current down over another period of 8 sec, which was repeated at the beginning and end of a 10 min period during REM sleep. Stimulation that lasts only a few seconds has been shown to produce a minimal effect on the brain, if any^98^. The aim of sham stimulation was to mimic the skin sensation that is occasionally experienced during the onset and offset of tDCS. This procedure is thought to make the two conditions subjectively indistinguishable^99^. The same procedure was repeated two or three times starting with the second sleep cycle.

### Electrophysiological recordings

To record EEG activity, 16 electrodes (Fp1, Fp2, F7, F3, Fz, F4, F8, T3, T4, T5, P3, Pz, P4, T6, O1, O2) were placed on the scalp following the standard 10–20 system^100^. C3, Cz and C4 electrode locations were left empty for the placement of tDCS electrodes. To record eye blinks and vertical saccades, two electrooculography (EOG) electrodes were placed below and above the left eye, while two other electrodes placed adjacent to the lateral canthi of each eye were used to measure horizontal saccades. An electromyography (EMG) electrode placed on the chin was used to record muscle tone, which was used for the scoring of sleep stages. The reference for all these electrodes was placed on the right ear mastoid and the ground electrode was placed on the temple. In addition, two bipolar EMG channels were used to record muscle activity in the right and the left arm flexor digitorum profundus, which were later used to analyze peripheral motor activity. Another two EMG channels recorded activity of the deltoid muscles in both arms. Electrophysiological recordings were continuously monitored on a computer screen and all electrodes were regularly checked throughout the night to ensure that the impedance remained under 5 kΩ. All data were recorded at 500 Hz sampling rate with Ag/AgCl electrodes using NeuroScan amplifier SynAmps Model 5083. Given that tDCS onset induces a slow frequency artifact in the EEG that may preclude online polysomnographic scoring, a 1-Hz high-pass filter was applied during recording for online monitoring of sleep stages^101^. As expected, tDCS onset- and offset-induced artifacts always faded away after 5–10 sec.

### Collection of dream reports

Based on previous research of tDCS over the motor cortex^97^, we expected the stimulation effects to last for at least one minute, providing a window of tDCS-artefact-free electrophysiological data. Thus, one minute after the termination of tDCS or sham-stimulation, participants were awakened from REM sleep with a standard awakening tone. They were then asked to give a verbal report of “everything that was going on in your mind before awakening”, aiming to facilitate dream recall. Afterwards, participants were asked if they remembered anything else about their dream. To avoid a possible bias between stimulation conditions, these questions were played from a pre-recorded computer audio file. Following the free dream report, participants were asked to fill in the BED Questionnaire. The questionnaire was designed as an internet survey programmed on www.webropol.com and was projected on a screen above the bed in the sleep laboratory. The monitor was on a flexibly moving arm and could easily be moved into a comfortable position, enabling participants to navigate and respond to the BED Questionnaire by controlling a mouse while lying in bed. To answer the questions with an open format, participants used a keyboard that was placed nearby.

Participants were stimulated and awakened two or three times per night, depending on how many REM sleep periods they had. The number of awakenings was balanced across the first and the second night and across the two stimulation conditions. White dream reports (i.e. cases when a person reports the occurrence of dream experiences but cannot recall any specific details) as well as sleep mentation reports (i.e. when a person reports non-perceptual subjective experiences, such as thinking) were excluded from the analysis. Two white dream and three sleep mentation reports, reported by five different participants, were excluded, as well as the answers to the BED Questionnaire (see below) collected after these verbal reports. All excluded reports were collected after the first awakening, and four of them after sham-stimulation. A total of 50 dreams reported during a total of 20 nights were available for analyses.

### Bodily Experiences in Dreams (BED) Questionnaire

The (maximally) 41-item BED Questionnaire was designed to gather detailed information about kinaesthetic and other bodily experiences in dreams. The BED Questionnaire consists of 5 general questions with movement sub-scales accessed through conditional branching (see Table 3; for a full list of questions, see Appendix 1). Each of the general questions targets a particular category of body-related experience: vestibular sensations, tactile and somatosensory experiences, movements, movement alterations, and body scheme alterations. Each general question, if and only if answered positively, is followed by sub-scales targeting more specific instances of this category of experience. For example, if a participant indicated that they had experienced movement sensations, they would then be asked about the occurrence of specific types of movement sensations, such as single, repetitive, and passive movements. In addition, if and only if participants reported specific sensations, they were additionally asked whether this sensation concerned the whole body, the right or left hand, the right or left side of the face, or another body part; if the latter was the case, they could indicate which one in open questions. If one of the main scales was answered negatively, the questionnaire skipped to the next general category without showing the subscales. Depending on whether a sub-scale asked about the intensity or the duration of experience, 9 point Likert-scales for answering ranged either from “1 = Low intensity” to “9 = High intensity” or from “1 = Never” to “9 = Throughout”.

**Table 3:** The BED Questionnaire: General questions and exemplary sub-scales.

*Five general questions (Yes/No)*
*Movement sub-scales (from 1* = *Never to 9* = *Throughout)*
5. Did you experience any tactile or somatosensory sensations in your dream?
11. Did you experience any vestibular or balance sensations in your dream?
14. Did you move in your dream (including active as well as passive movements (for instance in a vehicle) of the whole body or body parts)?
18. Were your movements (either of the whole body or of certain body parts) altered or impaired compared to wakefulness?
26. Was your dream body or were certain body parts altered compared to wakefulness?
15. How frequently did you move in your dream (including active as well as passive movements (for instance in a vehicle) of the whole body or body parts)?
16. How frequently did you perform the following types of movements in your dream?
16.1 – single actions (e.g. placing a book on the table)
16.2 – repetitive actions (e.g. running)
16.3 – passive movements (e.g. going by car)

While the BED Questionnaire was certainly demanding, it aimed to strike a balance between maximizing the specificity of questions, which was necessary to obtain sufficiently detailed data on dream experience, and minimizing complexity for participants and the time spent answering the questions before they could go back to sleep. We aimed to achieve this by designing the BED Questionnaire in a hierarchical and interactive way, meaning that participants first had to rate sensations on the five main scales and only in case of reporting the respective sensations, further sub-questions were shown to the participants.

While the five general categories of bodily experience were as inclusive as possible, for the specific questions for each category, we decided to focus on those body parts where we most expected to see an effect. As we stimulated over the hand and face areas - which, again, were chosen for their comparatively large size and the larger spread between left and right side cortical representation^102^ - we focused our questions on those areas. We also included open questions where participants could add details, for example about other body parts. We note that this option was rarely used by the participants (only 2.8% of the possible answers) and so was not further analysed.

We also note that this trade-off between the required specificity of phenomenological data and participant demand is not specific to our study, but exists, to some extent, for all dream studies using free reports and/or specific questionnaires following timed awakenings throughout the night. We believe that the fact that our questionnaire did show specific differences between conditions is heartening in this regard. We also note that previous research suggests that where a study targets specific aspects of dream phenomenology, such as emotions or bodily experiences, responses to a questionnaire may be more detailed than free dream reports, which often lack focus on the specific aspect targeted in a given study^50,51^. Therefore, while the questionnaire was certainly demanding, we believe it facilitated focus on those aspects we were most interested in. We also note that participants underwent training and were familiar with the questionnaire prior to the experimental nights: they were instructed to go through the online BED Questionnaire once during daytime in order to get familiar with all questions, and once after a spontaneous morning awakening at home, reporting their dream. They were also given the opportunity to ask any questions before the first laboratory night began.

Finally, one worry might be that participants freshly awakened from sleep might be particularly challenged by a detailed questionnaire asking them to reflect on specific aspects of dream phenomenology. However, we note that sleep inertia is largest after awakening from slow wave sleep^103^, whilst all awakenings in our study took place from REM sleep. Furthermore, one reason why we asked participants to give a free verbal report before responding to the questionnaire was to help jog their memories. Thus, any effects of sleep inertia were arguably lower when responding to the questionnaire than during the verbal reporting of dream experiences.

### Word count of verbal dream reports

The length of dream reports was assessed by two blind judges (authors J.W. and K.V.), who independently calculated the meaningful word count of each dream report. Murmurs, repetitions of words, and any secondary reflections or comments about the dream were not included in the word count. The judges initially agreed on the word count of 47 out of 50 dream reports (94% agreement). The judges discussed the reasons for the mismatch in the remaining 3 cases and reached an agreement.

### Content analysis of movement sensations in verbal dream reports

Following the findings from the BED Questionnaire, we carried out supplementary content analysis of verbal dream reports, focusing on the specific types of movement (single actions, repetitive actions, passive movement) performed by the dream self. To compare the type and frequency of movements reported in the BED Questionnaire to those explicitly mentioned in dream reports, two blind judges (authors V.N. and B.L.) carried out a content analysis of verbal reports. First, the judges scored whether each dream report contained at least one movement produced by the dream self, excluding facial movements such as talking, drinking, and blinking, as we reasoned that these would not typically be reported as movements (at least without explicit prompting or instruction). Movements attributed to the first-person plural “we” were treated as involving movements of the dream self. Second, in each dream that, in the first step, was judged to contain movement, the judges identified individual movements produced by the dream self as opposed to movements performed by other dream characters. Third, they scored the type of the identified movements (single action, repetitive action, passive movement). All three stages of content analysis were first carried out individually and the obtained results were then compared between the judges. In the case of disagreement, the judges discussed it until an agreement was achieved.

Regarding the presence or absence of movement in a given report, the judges initially agreed on 45 out of 50 dream reports (90% agreement). After discussion, the judges agreed that the remaining 4 dreams contained references to movements produced by the dream self, while one report contained no explicit references to such movement. Regarding individual movements, judges initially agreed on the identification of 33 movements and disagreed on 19 movements (63.5% agreement). The disagreement was caused by one judge either missing a movement or treating it as part of a longer sequence of movements, e.g. treating walking from A to B and from B to C as a single movement. This happened when movement descriptions in dream reports allowed for differing interpretations, leaving open whether they were associated with e.g. one or two distinct movements. By contrast, we speculate that this difference would have been more obvious to participants, as their responses to the questionnaire were based on their memory of the dream experience itself rather than a short and likely selective description of it, as is the case in the content analysis of dream reports. While we can only speculate about this point, this disagreement might reflect the greater suitability of our questionnaire to such detailed phenomenological questions as compared to content analyzing free reports.

After discussion, the judges agreed that the dream reports contained a total of 48 individual movements executed by the dream self. Regarding specific types of movements (single action, repetitive action, passive movement), the judges initially agreed on 44 out of 48 movements (91.7% agreement). After discussion, the judges agreed that the remaining 4 movements should be scored as follows: “diving” - single action, “riding a bike downhill” - passive movement, “writing something” - repetitive action, “made some coffee” - single action.

### EEG analysis: coherence and spectral power

To assess the electrophysiological effects of tDCS on brain functioning, we analyzed the full period of 1 min of EEG signal recorded between the termination of tDCS or sham-stimulation and controlled awakening. tDCS artifacts did not contaminate this EEG interval whilst sleep scoring ensured that REM sleep continued up to the point of awakening. On one occasion, a spontaneous awakening took place before the planned controlled awakening, and only 7 sec of stimulation-free sleep EEG were available for analysis. On another occasion, a spontaneous awakening took place immediately after the termination of stimulation; this recording was excluded, leaving 49 EEG recordings available for analysis.

Continuous recordings were first high-pass (0.5 Hz) and then low-pass (45 Hz) filtered using a FIR filter as implemented in EEGlab toolbox^104^. The data were then common average referenced, and excessively noisy periods of recording were manually deleted (an average of 743 ms per single recording). Detached or excessively noisy channels were deselected (an average of 0.2 channels per dataset), and an independent component analysis (ICA) was carried out on the remaining channels, using EEGlab toolbox^104^. Independent components reflecting eye movements and other sources of noise were manually deleted (an average of 3.3 ICs per recording), following which dropped noisy EEG channels were interpolated using spherical spline interpolation. Continuous EEG recordings were epoched into 2-sec segments with a 50% overlap between adjacent segments. Several epochs that still contained visible artifacts were manually deleted (an average of 0.5 epochs per recording). Individual epochs were demeaned across the whole 2 sec interval.

We analyzed EEG inter-hemispheric coherence in the beta oscillation range (15–30 Hz) at the electrodes adjacent to the stimulation site (F3, F4, T3, T4, P3, P4). Magnitude-squared coherence was computed in the range from 1.95 Hz to 44.92 Hz with a maximum frequency resolution of 1.95 Hz between pairs of EEG channels adjacent to the stimulation site from the frontal (F3-F4), temporal (T3-T4) and parietal (P3-P4) side, using Brainstorm toolbox^105^. Coherence values obtained at a single 2 sec segment level were averaged across beta frequency range (15.6–29.3 Hz). Next, coherence values were averaged across each 1-min pre-awakening recordings. Afterwards, individual means were averaged over several awakenings for each participant according to the experimental condition, yielding 10 tDCS and 10 sham-stimulation values for each electrode pair.

In the case of a significant difference between tDCS and sham-stimulation conditions across the 1-min pre-awakening periods, coherence was computed at four separate 15 sec sub-intervals preceding controlled awakening: −60 to −46 sec, −45 sec to −31 sec, −30 to −16 sec, and −15 to −1 sec. A significant difference between tDCS and sham conditions observed immediately after the termination of stimulation (−60 to −46 sec) was expected to reflect a tDCS-driven modulation of EEG activity, as an effect size of neurophysiological changes following motor tDCS decreases with increasing time^97^. Contrary to this, a significant difference between tDCS and sham-stimulation conditions at the interval preceding awakening (−15 to −1 sec) with no difference at the −60 to −46 sec interval was expected to reflect an unspecific modulation of EEG activity, e.g. micro-arousals caused by tingling sensations could eventually trigger body movements in bed.

To control for a possible confound of EEG spectral power on coherence computation (Bowyer, 2016), we carried out a control analysis of EEG beta power. Spectral power was computed across 2 sec epochs using Hilbert transform, set from 1 Hz to 44 Hz in steps of 1 Hz, for the same set of 6 electrodes adjacent to the stimulation site. Power values obtained at a single 2 sec segment level were averaged across beta frequency range (15–30 Hz), with subsequent data averaging steps repeating coherence analysis.

Finally, as an exploratory data analysis, inter-hemispheric EEG coherence was compared between sham-stimulation and tDCS conditions for each individual frequency across the whole 1.95–44.92 Hz range (see Supplementary Material and Figs. S1 and S2).

### Phasic EMG analysis

We investigated the effects of tDCS on peripheral muscle tone by analyzing EMG activity from the left/right arm flexor and deltoideus muscles during the 1 min interval between the termination of tDCS or sham-stimulation and the controlled awakening of participants. We were specifically interested whether EMG traces following tDCS and sham-stimulation showed increased phasic muscle activity compared to the pre-stimulation baseline window, and whether bihemispheric tDCS modulated interaction between the left/right arm EMG. Since phasic EMG activity manifests during REM sleep as short-lasting muscle bursts recorded by surface electrodes^106^, we split the 1-min epochs into 60 non-overlapping 1-sec segments and carried out a binary assessment whether each segment contained phasic EMG activity. Segments with phasic EMG activity were then assigned a value of one, and segments without phasic EMG activity a value of zero. The mean overall 60 binary values were then used to define the ratio of phasic EMG activity within the respective epoch.

**Figure 5:**
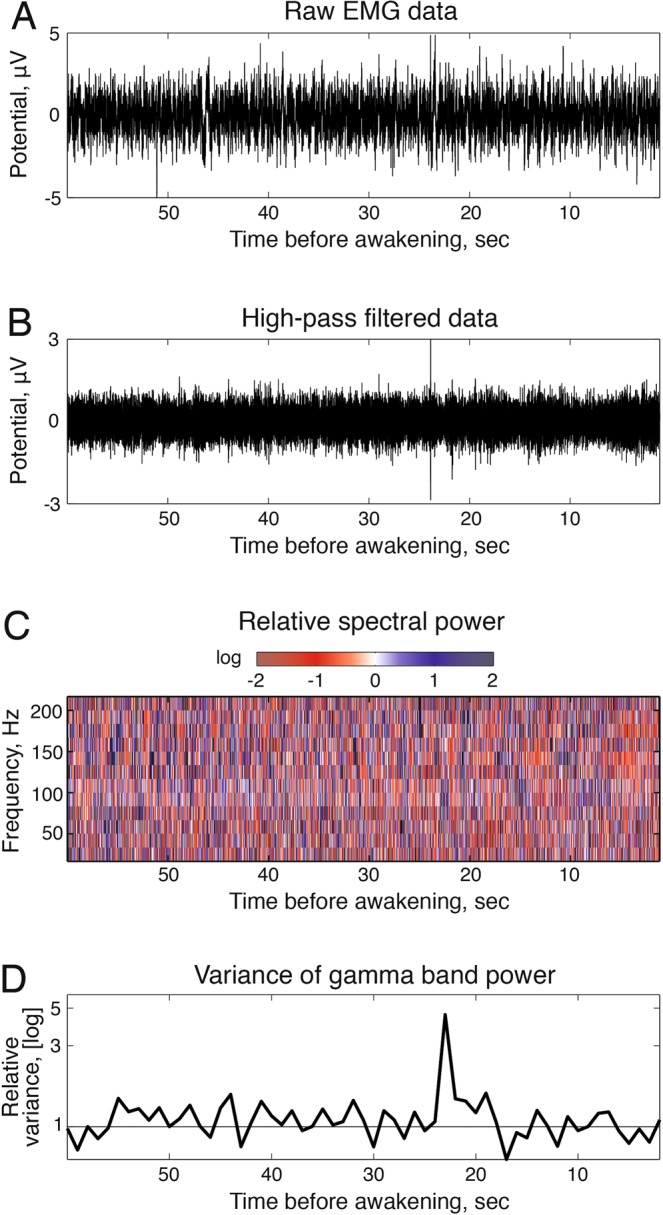
Analysis of peripheral EMG activity. (A) Exemplary 60 sec EMG recording of the right hand flexoris muscle between termination of tDCS and the awakening. **(B)** The same EMG recording after a high-pass filter with a 50 Hz cutoff-frequency. **(C)** Relative spectral power of the high-pass filtered EMG recording. **(D)** Relative variance of gamma band power, i.e., divided by the average variance of gamma band power in the baseline time window. Values greater than one (above the grey solid line) depict 1 sec segments with a shift towards phasic EMG, values equal or smaller than one depict segments with a shift towards tonic EMG.

More specifically, since phasic EMG activity is reflected in broadband spectral power changes, we used the variance of gamma band (50–250 Hz) power for the detection of short-lasting bursts of muscle activity. In a first step we high-pass filtered the raw data with a 3rd order butterworth filter with a cutoff-frequency at 50 Hz (Fig. 5A,B). For the subsequent time-frequency analysis, we used a single-tapered spectral analysis method^107^ with a time window of 50 ms and 10-ms time steps. The relative power changes were then calculated by dividing the time-resolved amplitude for each frequency bin by the frequency-specific average of the whole 1 min epoch (Fig. 5C). After splitting the epochs in 1-s segments, the variance of relative power was calculated for each segment and every frequency. The variance of gamma band power was then defined as the mean over all frequencies between 50 Hz and 250 Hz.

To assess a relative shift towards more phasic/tonic activity in response to stimulation, the variance of gamma band power was calculated both for the 60 sec epochs after the termination of tDCS or sham-stimulation and for a 30 sec baseline time window before tDCS or sham-stimulation. The relative variance of gamma band power was then calculated by dividing the variance by the averaged variance in the baseline time window (Fig. 5D). This way, post-stimulation segments with the variance of gamma band power higher than the corresponding average (median) in the stimulation-free 30 sec baseline time window received a relative variance value greater than one and were defined as segments shifting towards phasic EMG, while segments with a relative variance between zero and one were defined as segments shifting towards tonic EMG. Finally, a proportion of phasic segments was calculated across the whole 60 sec post-stimulation epoch, yielding values ranging from 0, indicating a complete shift towards tonic EMG, to 1, indicating a complete shift towards phasic EMG.

### Statistical analysis

All dependent measures were averaged per individual participant separately for the sham-stimulation and tDCS conditions. A Shapiro-Wilk test was used to assess the distribution normality of dependent variables. Paired-samples t test and Pearson correlation were carried out when distribution of given variables (or their difference in a case of t test) was normal, and Wilcoxon signed-rank test (Z statistic) and Spearman rank order correlation were used in the cases of non-normal distribution of one or both variables. For the paired-samples t-test, Cohen’s d was calculated as an effect size estimate using pooled variance. For the Wilcoxon signed-rank test, r = Z/sqrt(N) was calculated as an effect size estimate. All statistical tests were two-tailed. To control for multiple comparisons, Bonferroni correction was applied by multiplying the obtained p value by the number of comparisons with a given set of tests. Bonferroni corrected p values are denoted as p_BxN_ where N indicates the number of multiple comparisons. For all control analyses, we report uncorrected p values. For the control t tests where we expected null findings, we additionally report Bayes factor in favor of the null. Statistical analyses were carried out with SPSS 22 and JASP 0.8.2.

## Supplementary information

Supplementary Material.

## Data Availability

The datasets analyzed during the current study are available from the corresponding author on reasonable request.
