# Supplementary material for: Modulating dream experience: Noninvasive brain stimulation over the sensorimotor cortex reduces dream movement
Source: Sci Rep. 2020 Apr 21;10:6735. doi: 10.1038/s41598-020-63479-6 (PMC7174293; doi:10.1038/s41598-020-63479-6)
Supplement: Supplementary file 1 — Supplementary Material. [file 41598_2020_63479_MOESM1_ESM.pdf]

Modulating dream experience: Noninvasive brain stimulation over the sensorimotor cortex reduces dream movement

Valdas Noreika, Jennifer M. Windt, Markus Kern, Katja Valli, Tiina Salonen,

Riitta Parkkola, Antti Revonsuo, Ahmed A. Karim, Tonio Ball, Bigna Lenggenhager

SUPPLEMENTARY MATERIAL

Supplementary content analysis of movement sensations in verbal dream reports

Movements were reported in 49.8% (SEM=10) of dreams following sham-stimulation and 54.9% (SEM=10.9) of dreams following tDCS. Repetitive actions were the most common type of movement, followed by single actions, with passive movements being the least common (see Tables 2 and S1), replicating the pattern observed in the BED Questionnaire data. However, there were no significant differences between the sham-stimulation and tDCS conditions (see Table S1), in contrast to the effects observed in the questionnaire data (see Table 1). The discrepancy could be due to a considerably smaller proportion of explicitly expressed movements in free dream reports compared to the BED Questionnaire answers, i.e. participants tended to omit explicit descriptions of movements from the spontaneous verbal reports.

Nevertheless, the proportion of repetitive actions correlated strongly between the free dream reports and the BED Questionnaire answers in the sham-stimulation condition (Spearman rank order correlation: rho=0.81, p_{B-8}=0.033), indicating a strong convergence between these two types of measurement. Interestingly, this association did not hold in the tDCS condition (rho=-0.19, p_{B-8}=1). No other correlations were significant.

Supplementary Table S1. Dream content analysis: Percentage of dream reports containing movement sensations following sham-stimulation and tDCS during REM sleep

	Sham	*tDCS*	*Statistical test*	
	M (SEM)	*M (SEM)*	*t/Z*	*p*
Movement				
	49.8 (10)	54.9 (10.9)	t(9) = 0.31	0.77
Movement sub-scales				
Single actions	21.6 (10.5)	24.9 (8.6)	t(9) = 0.19	0.85
Repetitive actions	38.2 (12.7)	43.2 (9.4)	Z = 0.54	0.59
Passive movements	16.6 (7)	8.3 (5.7)	Z = 0.76	0.45

Note. t: paired samples t test; Z: Wilcoxon signed-rank test. Uncorrected p values.

Control analysis of the time of night effect on the presence of bodily experiences in dreams

To control the possible effect of the time of night on the frequency of bodily experiences reported in the BED Questionnaire upon awakening, we compared the answers to the five main questions regarding the presence of different types of bodily experiences (tactile-somatosensory, vestibular-balance, movement, movement alterations, body scheme alterations) between the first and the last reports of each night, separately for the sham-stimulation and tDCS conditions. Cases in which a given experience was present in the first but not in the last set of questionnaire responses from the same night were coded as -1. Cases in which a given experience was either present or absent in both the first and the last set of questionnaire responses from the same night were coded as 0, indicating no change during night. Finally, cases in which a given experience was absent in the first but present in the last set of questionnaire responses from the same night were coded as 1. We observed no systematic difference between the first and the last set of questionnaire responses from the same night, excluding cumulative effects over night (see Supplementary Table S1).

Supplementary Table S2. Relative values on a Likert scale comparing answers to the BED Questionnaire between the first and the last report within the same night

Participant ID	*5. Tactile-somatosensory*		*11. Vestibular-balance*		*14. Movement*		*18. Movement alterations*		*26. Body scheme alterations*	
	Sham	*tDCS*	*Sham*	*tDCS*	*Sham*	*tDCS*	*Sham*	*tDCS*	*Sham*	*tDCS*
1	-1	0	0	0	0	0	0	0	0	0
2	0	0	0	0	1	1	0	0	0	0
3	0	1	0	0	0	0	0	1	0	0
4	-1	0	0	0	0	0	-1	0	0	0
5	-1	0	-1	0	0	0	0	0	0	0
6	1	-1	0	0	0	1	1	0	0	0
7	0	1	0	0	0	-1	0	0	0	0
8	0	1	0	0	-1	0	-1	0	-1	0
9	0	0	0	0	0	-1	0	0	0	0
10	0	0	-1	0	0	0	0	0	0	0

tDCS modulation of EEG coherence: Exploratory analysis of individual frequencies

While hypothesis-based analysis was carried out by averaging EEG beta coherence over 15-30 Hz frequency range, exploratory pairwise comparisons across a wider range of frequencies (2-45 Hz) indicated that the tDCS effect was maximal between parietal electrodes P3 and P4 in the high beta range (21-31 Hz) ($t(9)$ range=[2.29 3.23], p range=[0.01 0.048] (uncorrected), Cohen's d range=[0.84 0.93]) (see Fig. S1). Contrary to this, there were no tDCS effects in the delta, theta, alpha and gamma frequency ranges (see Fig. S1). Likewise, tDCS did not produce a significant modulation of inter-hemispheric coherence between the pairs of frontal (min/max $t(9)=1.18$, $p=0.27$ (uncorrected), Cohen's $d=0.52$) or temporal (min/max $t(9)=-1.16$, $p=0.28$ (uncorrected), Cohen's $d=0.52$) electrodes adjacent to the stimulation site (see Fig. S1)..

A more fine-grained analysis of four 15 sec time windows between the termination of stimulation and controlled awakenings indicated a short-lasting decrease of parietal coherence in the low gamma frequency range (37-43 Hz) immediately following tDCS, i.e. from -60 to -31 sec before awakenings (min/max $t(9)=2.72$, $p=0.024$ (uncorrected), Cohen's $d=1.18$; see Fig. S2).

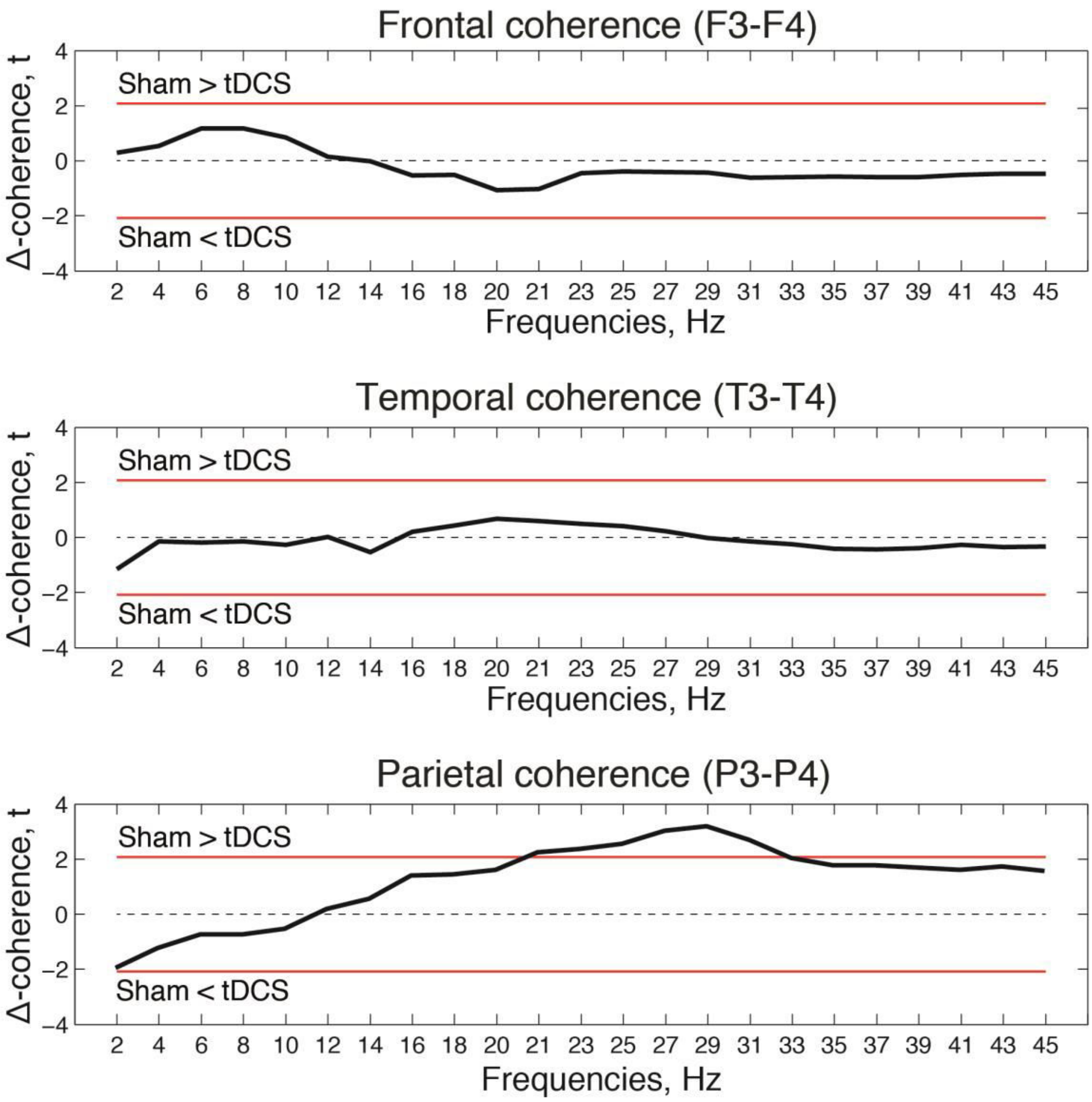

Figure S1 | Exploratory analysis of inter-hemispheric EEG coherence. Inter-hemispheric EEG coherence between frontal (top), temporal (middle), and parietal (bottom) electrodes surrounding the tDCS site, expressed on the y axis as t statistic of a contrast between sham-stimulation and tDCS conditions (Δ-coherence). A separate paired samples t test was carried out for each individual frequency from 1.95 Hz to 44.92 Hz separated in steps of 1.95 Hz, calculated over the whole 60 sec period from the termination of stimulation to the controlled awakenings. Individual frequencies are represented on the x axis. Red lines depict the significance threshold (uncorrected $p<0.05$). Positive t values indicate higher coherence in the sham-stimulation condition, whereas negative t values indicate higher coherence in the tDCS condition.

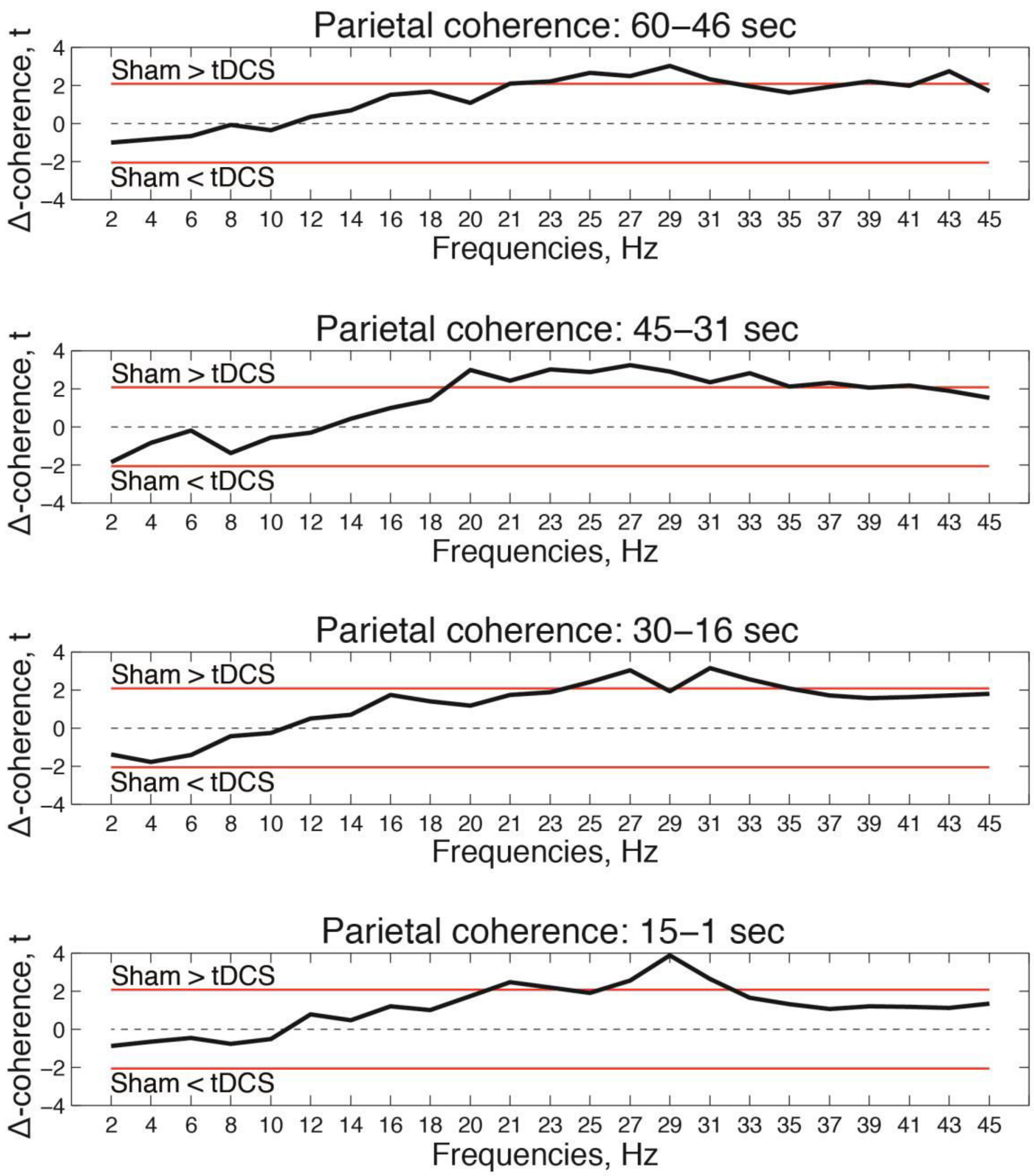

Figure S2 | Exploratory analysis of inter-hemispheric parietal EEG coherence one minute before the awakenings. Inter-hemispheric EEG coherence between parietal electrodes P3 and P4 located posterior to the tDCS site is depicted on the y axis as t statistic of a contrast between sham-stimulation and tDCS conditions (Δ-coherence). Δ-coherence is plotted separately in four stimulation-free time intervals preceding controlled awakenings from REM sleep. A separate paired samples t test was carried out for each individual frequency from 1.95 Hz to 44.92 Hz separated in steps of 1.95 Hz, which are represented on the x axis. Red lines depict the significance threshold (uncorrected $p<0.05$). Positive t values indicate higher coherence in the sham-stimulation condition, whereas negative t values indicate higher coherence in the tDCS condition.

Appendix 1

Bodily Experiences in Dreams (BED) Questionnaire

1) Subject's code, date, experimental condition, time of awakening

2) Do you think you remembered everything about your dream? *

- yes
- no

3) Did you omit, censor, or change certain parts of your dream in your dream report? *

- yes
- no

4) Describe how you perceived the dream *

Please only mark one option in each horizontal line!

	never	..2..	..3..	..4..	..5..	..6..	..7..	..8..	throughout
I watched the dream from the outside, as if on a screen *	€	€	€	€	€	€	€	€	€
I was present in the dream, but as a passive observer *	€	€	€	€	€	€	€	€	€
I was present in the dream, as an active participant *	€	€	€	€	€	€	€	€	€

5) Did you experience any tactile or somatosensory sensations in your dream? *

(for instance touching something, being touched, self-touch, cold, heat, pleasant or unpleasant bodily sensations, numbness, vibration, tickling, pain, pressure, or itching)

yes no

6) Please specify the duration, body part localization and intensity of tactile and somatosensory sensations in your dream! *

	never	..2..	..3..	..4..	..5..	..6..	..7..	..8..	through out	whole body	right hand	left hand	right side of face	left side of face	other body part	low intensity	..2..	..3..	..4..	..5..	..6..	..7..	..8..	high intensity
*	€	€	€	€	€	€	€	€	€	€	€	€	€	€	€	€	€	€	€	€	€	€	€	€

For each sensation, mark the duration, whole body / body part and intensity. If you mark "never", jump to the next sensation.

7) Did you experience any of the following sensations? *

	never	..2..	..3..	..4..	..5..	..6..	..7..	..8..	through out	whole body	right hand	left hand	right side of face	left side of face	other body part	low intensity	..2..	..3..	..4..	..5..	..6..	..7..	..8..	high intensity
touching something *	€	€	€	€	€	€	€	€	€	€	€	€	€	€	€	€	€	€	€	€	€	€	€	€
being touched *	€	€	€	€	€	€	€	€	€	€	€	€	€	€	€	€	€	€	€	€	€	€	€	€
self-touch *	€	€	€	€	€	€	€	€	€	€	€	€	€	€	€	€	€	€	€	€	€	€	€	€
cold *	€	€	€	€	€	€	€	€	€	€	€	€	€	€	€	€	€	€	€	€	€	€	€	€
hot *	€	€	€	€	€	€	€	€	€	€	€	€	€	€	€	€	€	€	€	€	€	€	€	€

For each sensation, mark the duration, whole body / body part and intensity. If you mark "never", jump to the next sensation.

8) Did you experience any of the following sensations? *

	never	..2..	..3..	..4..	..5..	..6..	..7..	..8..	through out	whole body	right hand	left hand	right side of face	left side of face	other body part	low intensity	..2..	..3..	..4..	..5..	..6..	..7..	..8..	high intensity
pleasant bodily sensations *	€	€	€	€	€	€	€	€	€	€	€	€	€	€	€	€	€	€	€	€	€	€	€	€
unpleasant bodily sensations *	€	€	€	€	€	€	€	€	€	€	€	€	€	€	€	€	€	€	€	€	€	€	€	€
numbness *	€	€	€	€	€	€	€	€	€	€	€	€	€	€	€	€	€	€	€	€	€	€	€	€
vibration *	€	€	€	€	€	€	€	€	€	€	€	€	€	€	€	€	€	€	€	€	€	€	€	€
tickling *	€	€	€	€	€	€	€	€	€	€	€	€	€	€	€	€	€	€	€	€	€	€	€	€

For each sensation, mark the duration, whole body / body part and intensity. If you mark "never", jump to the next sensation.

9) Did you experience any of the following sensations? *

	never	..2..	..3..	..4..	..5..	..6..	..7..	..8..	through out	whole body	right hand	left hand	right side of face	left side of face	other body part	low intensity	..2..	..3..	..4..	..5..	..6..	..7..	..8..	high intensity
pain *																								
pressure *																								
itching *																								

10) If you experienced other sensations and/or marked "other body part", please specify which sensation, which body part and on which side of the body (right / left / both)!

11) Did you experience any vestibular or balance sensations in your dream? *

(for instance falling, floating, flying, spinning, or strange movement trajectories)

yes no

12) Please specify the duration of vestibular and balance sensations in your dream! *

	never	..2..	..3..	..4..	..5..	..6..	..7..	..8..	throughout
*									

13) Did you experience any of the following vestibular sensations in your dream? *

Please only mark one option in each horizontal line!

	never	..2..	..3..	..4..	..5..	..6..	..7..	..8..	throughout
falling *									
floating *									
flying *									
spinning *									
strange movement trajectories (back and forth, curvilinear) *									

14) Did you move in your dream (including active as well as passive movements (for instance in a vehicle) of the whole body or body parts)?

yes no

15) How frequently did you move in your dream (including active as well as passive movements (for instance in a vehicle) of the whole body or body parts)?

	never	..2..	..3..	..4..	..5..	..6..	..7..	..8..	throughout
*									

16) How frequently did you perform the following types of movements in your dream?

	never	..2..	..3..	..4..	..5..	..6..	..7..	..8..	through out	whole body	right hand	left hand	right side of face	left side of face	other body part
single actions (e.g. placing a book on the table) *															
repetitive actions (e.g. running) *															
passive movements (e.g. going by car) *															

17) If you marked "other body part", please specify which body part and on which side of the body (right / left / both)!

18) Were your movements (either of the whole body or of certain body parts) altered or impaired compared to wakefulness? *

(for instance enhanced or reduced precision, enhanced or reduced coordination, physically impossible movement, enhanced effort, effortlessness, immobility, paralysis, forced or prevented movement, repetitive, slowed or hurried movement, acceleration)

yes no

19) Please specify the duration and body part localization of different types of altered or impaired movements in your dream! *

	never	..2..	..3..	..4..	..5..	..6..	..7..	..8..	through out	whole body	right hand	left hand	right side of face	left side of face	other body part
*															

20) Did you experience any of the following types of altered or impaired movements in your dream? *

For each movement, mark the duration and body part. If you mark "never", jump to the next movement.

	never	..2..	..3..	..4..	..5..	..6..	..7..	..8..	through out	whole body	right hand	left hand	right side of face	left side of face	other body part
enhanced precision *															
reduced precision *															
enhanced coordination *															
reduced coordination *															
physically impossible movement *															

21) Did you experience any of the following types of altered or impaired movements in your dream? *

For each movement, mark the duration and body part. If you mark "never", jump to the next movement.

	never	..2..	..3..	..4..	..5..	..6..	..7..	..8..	through out	whole body	right hand	left hand	right side of face	left side of face	other body part
enhanced effort *															
effortlessness *															
immobility *															
paralysis *															
forced movement *															
prevented movement (being held back or tied up) *															

22) Did you experience any of the following types of altered or impaired movements in your dream? *

For each movement, mark the duration and body part. If you mark "never", jump to the next movement.

	never	..2..	..3..	..4..	..5..	..6..	..7..	..8..	through out	whole body	right hand	left hand	right side of face	left side of face	other body part
repetitive *															
slowed *															
hurried / acceleration *															

23) If you experienced other types of altered or impaired movements and/or marked "other body part", please specify which type of movement, which body part and on which side of the body (right / left / both)!

24) How often were you aware of your dream body (either of the whole body or of certain body parts) in your dream? *

	never	..2..	..3..	..4..	..5..	..6..	..7..	..8..	through out	whole body	right hand	left hand	right side of face	left side of face	other body part
*															

25) If you marked "other", please specify!

26) Was your dream body or were certain body parts altered compared to wakefulness? *

(such alterations may include having supernumerary limbs or a doppelgänger (that is, the presence of a double in addition to your dream self), absent limbs or the complete lack of a body, heavier, lighter, bigger, smaller or animal body parts or whole body, being a different sex, being a different person of the same sex, being older, younger, being an object etc, loss of ownership for whole body or certain body parts)

yes no

27) Please specify the duration and body part localization of alterations to your dream body! *

	never	..2..	..3..	..4..	..5..	..6..	..7..	..8..	through out	whole body	right hand	left hand	right side of face	left side of face	other body part
*															

For each type of alteration, mark the duration and whole body / body part. If you mark "never", jump to the next sensation. A SUPERNUMERARY LIMB refers to the presence of an additional (physically impossible) body part. A DOPPELGÄNGER refers to the presence of a double in addition to the dream self (i.e., the character you are identifying with in your dream).

28) In what way was your dream body altered compared to your waking body? *

	never	..2..	..3..	..4..	..5..	..6..	..7..	..8..	through out	whole body	right hand	left hand	right side of face	left side of face	other body part
supernumerary limb/doppelgänger *															
absent *															
heavier *															
lighter *															
bigger *															

For each type of alteration, mark the duration and whole body / body part. If you mark "never", jump to the next sensation.

29) In what way was your dream body altered compared to your waking body? *

	never	..2..	..3..	..4..	..5..	..6..	..7..	..8..	through out	whole body	right hand	left hand	right side of face	left side of face	other body part
smaller *															
animal *															
different sex *															
different person, same sex *															
older *															

For each type of alteration, mark the duration and whole body / body part. If you mark "never", jump to the next sensation.

30) In what way was your dream body altered compared to your waking body? *

	never	..2..	..3..	..4..	..5..	..6..	..7..	..8..	through out	whole body	right hand	left hand	right side of face	left side of face	other body part
younger *															
being an object *															
felt alien or as if it belonged to someone else *															
changed in some other way *															

31) If you marked "other body part" or "changed in some other way", please specify which type of alteration, which body part and on which side of the body (right / left / both)!

32) How did you know or perceive that your body was changed? *

Multiple answers to this question are possible!

- just knew
- saw it
- felt it
- heard it
- other

33) If you marked "other", please specify!

34) Did you identify with more than one dream character, that is, did you have the feeling that you actually turned into another dream character during your dream, or that you had more than one dream self? *

Please indicate the duration and, if applicable, whether you identified with more than one dream character simultaneously or consecutively, or whether you "just knew" this to be the case. If you mark never, please jump to the next question.

	never	..2..	..3..	..4..	..5..	..6..	..7..	..8..	throughout	simultaneously	consecutively	just knew
*												

Please mark the duration and indicate for which part of the body this was the case. If you mark never, please jump to the next question.

35) Did you experience your sleeping physical body during your dream? *

	never	..2..	..3..	..4..	..5..	..6..	..7..	..8..	throughout	whole body	right hand	left hand	right side of face	left side of face	other body part
*															

36) If you marked "other body part", please specify which body part and on which side of the body (right / left / both)!

For each sensation, please mark the duration and the intensity. If you mark never, please jump to the next line.

37) Did you experience any of the following *

	never	..2..	..3..	..4..	..5..	..6..	..7..	..8..	through out	low intensity	..2..	..3..	..4..	..5..	..6..	..7..	..8..	high intensity
nausea/dizziness *																		
heart beat *																		
breathing *																		
hunger *																		
thirst *																		

38) Did you experience any of the following

	never	..2..	..3..	..4..	..5..	..6..	..7..	..8..	through out	low intensity	..2..	..3..	..4..	..5..	..6..	..7..	..8..	high intensity
urge to use the toilet *																		
sexual excitement *																		
fatigue/exhaustion *																		
nudity *																		
being inadequately dressed *																		

39) Is there anything else that you would like to remark about your dream? Did you remember more about your dream than you initally reported in your free dream report? If so, please report these things below!

40) Did you omit, censor, or change certain parts of your dream in your answers to these questions? *

- yes
- no

41) Please try to estimate the duration of your dream. How long did your dream seem to last, according to subjective experience (in minutes)? *

- I want to submit my answers

Submit
